# Hypoxia-Inducible Factor-1α Gene (*HIF1A*) rs11549465 (1772 C>T) Polymorphism, Body Mass Index, Smoking, and Cutaneous Melanoma: An Observational Case–Control Study

**DOI:** 10.3390/cancers18172787

**Published:** 2026-08-27

**Authors:** Cinzia Buligan, Vincenzo Maione, Gianluca Petris, Giuseppe Stinco, Sabina Cauci

**Affiliations:** 1 Department of Medicine, University of Udine, Piazzale Kolbe 4, 33100 Udine, Italy; cinzia.buligan@asufc.sanita.fvg.it (C.B.); gianluca.petris@uniud.it (G.P.); giuseppe.stinco@uniud.it (G.S.); 2 Institute of Dermatology, Azienda Sanitaria Universitaria Friuli Centrale (ASUFC), Santa Maria della Misericordia, Friuli Centrale University Healthcare Hospital of Udine, 33100 Udine, Italy; 3 Dermatology Clinic, Department of Medical Sciences, University of Brescia, 25123 Brescia, Italy; vincenzo.maione@unibs.it; 4 Fondazione Italiana Fegato ETS—Italian Liver Foundation ETS, 34149 Trieste, Italy

**Keywords:** skin cancer, metastasis, head and neck cancer, genetic variant, single-nucleotide polymorphism, hypoxia, oxidative stress, cigarette smoke, obesity

## Abstract

Background: Hypoxia and oxidative stress are central features of cutaneous melanoma biology. Body mass index (BMI) and smoking, both of which can influence systemic hypoxia, inflammation, and oxidative stress, have shown inconsistent associations with melanoma. We investigated the association of the hypoxia-inducible factor-1 alpha gene (*HIF1A*) rs11549465 (1772 C>T; Pro582Ser) polymorphism, alone and in combination with overweight/obesity or smoking habits, with cutaneous melanoma susceptibility and clinicopathological characteristics. Methods: This observational case–control study included 132 Caucasian Italian patients with cutaneous melanoma and 312 healthy controls. The rs11549465 polymorphism was genotyped by genomic DNA restriction fragment analysis. Logistic regression provided age- and sex-adjusted and mutually adjusted estimates. Results: Genotype and allele frequencies did not differ between patients and controls. Within the melanoma cohort, smoking for ≥20 years remained associated with metastatic disease after adjustment for BMI ≥ 25 kg/m^2^, age at diagnosis, and sex (aOR = 3.12, *p* = 0.006), whereas BMI did not (aOR = 1.23, *p* = 0.612). Compared with healthy controls, metastatic melanoma was independently associated with BMI ≥ 25 kg/m^2^ (aOR = 2.26, *p* = 0.010) and smoking for ≥20 years (aOR = 3.85, *p* < 0.001). Mean pack-years were higher in melanoma patients than controls (9.4 ± 16.7 vs. 5.1 ± 12.2; *p* = 0.002), and ≥10 and ≥20 pack-years remained associated after age- and sex-adjustment (aOR = 2.19, *p* = 0.001 and aOR = 3.31, *p* < 0.001, respectively). Mean pack-years were higher in MetM than NMetM (13.1 ± 21.0 vs. 5.9 ± 10.0; *p* = 0.013), and ≥20 pack-years was associated with MetM (OR = 3.05, *p* = 0.017). Adjusted associations with head/neck melanoma (n = 13) were observed for CC plus BMI ≥ 30 kg/m^2^ (aOR = 9.07, *p* < 0.001), ≥20 cigarette/day (aOR = 5.96, *p* = 0.004), ≥20 years smoking (aOR = 6.64, *p* = 0.003), and other smoking measures. Conclusions: To our knowledge, this is the first report on the interplay between the rs11549465 polymorphism and cutaneous melanoma. *HIF1A* rs11549465 was not independently associated with melanoma susceptibility. Associations involving smoking, BMI, joint genotype–lifestyle exposures, and anatomical localization had wide confidence intervals and were exploratory; small subgroups and multiple comparisons require cautious interpretation and independent validation.

## Simple Summary

Cutaneous melanoma may be influenced by genetic background and lifestyle-related hypoxia and oxidative stress. We examined *HIF1A* rs11549465 (C>T) in 132 Italian patients with cutaneous melanoma and 312 healthy controls. The variant alone was not associated with melanoma susceptibility. Within the melanoma group, smoking for ≥20 years remained associated with metastatic disease after adjustment for body mass index (BMI), age at diagnosis, and sex. Compared with healthy controls, both BMI ≥ 25 kg/m^2^ and smoking for ≥20 years remained independently associated with metastatic melanoma. Head/neck melanoma showed higher frequencies of the rs11549465 CC genotype combined with obesity and several smoking-related exposures than melanomas at other sites and healthy controls. Because some subgroup analyses involved small numbers and multiple correlated comparisons, these findings should be considered exploratory. Larger independent studies are needed to establish whether the *HIF1A* genotype modifies the effects of body weight or smoking on melanoma development/progression and localization.

## 1. Introduction

Cutaneous melanoma is an aggressive skin malignancy with a marked propensity for metastasis and substantial mortality [1,2,3]. Since the 1990s, its global burden has increased steadily, with considerable variation across ethnic groups and geographical regions. Incidence is highest in predominantly fair-skinned populations, particularly in Western Europe, Australasia, and high-income North America [1]. In Italy, melanoma accounts for approximately 5% of newly diagnosed cancers. For 2025, the estimated age-standardized incidence rates are 29.0 per 100,000 men and 25.4 per 100,000 women. Compared with 2013–2017, incidence is projected to increase by 29% overall and by 44% among women [4,5].

Modern immunotherapies and targeted treatments have substantially improved survival in advanced melanoma; nevertheless, 40–60% of patients still experience disease recurrence [2,3]. These treatments can also produce clinically important adverse effects. More effective risk stratification and individualized management strategies are therefore needed [2,3,6].

The marked clinical heterogeneity of cutaneous melanoma supports the search for additional biomarkers that could refine risk assessment and disease stratification [6]. Tumor thickness, ulceration, mitotic activity, lymph node involvement, and distant metastasis remain essential prognostic parameters, but patients with similar clinicopathological features may follow different clinical courses [6]. Molecular, genetic, metabolic, and immune determinants may therefore contribute to melanoma susceptibility and progression. Germline variants affecting inflammation, immune surveillance, oxidative stress, and hypoxia responses may help explain inter-individual differences and could inform preventive strategies in people exposed to environmental or lifestyle-related risk factors [6,7,8,9].

Established or proposed melanoma risk factors include ultraviolet (UV) radiation, indoor tanning, family history, fair skin, immune dysregulation, vitamin D-related pathways, environmental pollution, smoking, obesity, and oxidative stress [1,2,3,6,7,8,9,10,11,12,13,14,15,16,17]. Somatic alterations in genes such as BRAF and NRAS are central to melanoma pathogenesis [18], and germline polymorphisms in immune- and vitamin D-related genes have also been associated with melanoma [13,14,15,19,20,21,22].

A substantial proportion of the inherited component of melanoma susceptibility remains unexplained [22]. Following the identification of CDKN2A as a major susceptibility gene, genome-wide association studies identified additional loci, including immune-related variants at the HLA locus and the rs408825 variant, associated with expression of the innate immunity gene MX2 [22].

These observations support the further investigation of genetic variants involved in immune regulation, oxidative stress, and hypoxia. Predicting individual risk of developing melanoma may help in the implementation of effective risk-reduction measures [6,8,9].

Oxidative stress and hypoxia are closely interconnected with melanoma biology; indeed, nutrient-based antioxidant approaches have been proposed for prevention and adjunctive therapy [17,23,24]. Melanocytes are physiologically exposed to pro-oxidant stimuli, including UV radiation, melanin synthesis, inflammatory mediators, and environmental pollutants, which can generate reactive oxygen species that promote DNA damage, altered signaling, and tumor-promoting inflammation [17,23,24]. Smoking and excess body weight may further contribute to oxidative, inflammatory, and metabolic stress [17,23,24,25,26]. These exposures may therefore be relevant when genetic variation in hypoxia-response pathways is investigated in melanoma.

Hypoxia-inducible factor-1 alpha (HIF-1α) is a major regulator of cellular adaptation to reduced oxygen availability, including the hypoxic conditions present in solid tumors [27]. HIF-1α, after dimerization with the constitutively expressed HIF-1β, forms the HIF-1 transcription factor, which regulates the expression of thousands of genes involved in angiogenesis, including upregulation of the vascular endothelial growth factor (VEGF) expression, glucose metabolism, cell differentiation, and adaptation to metabolic stress [27,28,29,30,31,32]. HIF-1, together with the related factor HIF-2, regulates genes that have roles in the progression of several tumors [27,30,31], and therefore the modulation of HIFs has been proposed as a new approach in cancer therapy [30,31,32].

Within the tumor microenvironment, hypoxia actively drives malignant progression [27,28,29,30,31,32] by stabilizing HIF-1α and promoting metabolic adaptation, angiogenesis, resistance to apoptosis, invasion, and extracellular matrix remodeling [27,30,31,32]. These processes are relevant to melanoma, as they may foster immune suppression and weaken antitumor responses [27,30,31,32,33,34], potentially reducing the efficacy of immune checkpoint inhibitors [27,28,29,30,31,32,33,34]. HIF-1α is constitutively expressed in melanoma cells, and its elevated expression has been correlated with melanoma aggressiveness [35]. Interestingly, treatment with acriflavine, an inhibitor of HIF-1α, has been shown to provoke consistent melanoma cell death [36].

Among the known coding variants of the HIF-1α gene (*HIF1A*), rs11549465 (1772 C>T) polymorphism was selected for the present study because it is a non-synonymous polymorphism producing a Pro582Ser amino-acid substitution and has reported functional effects on HIF-1α transcriptional activity [37,38,39]. Thus, unlike variants whose biological relevance is primarily inferred from genetic association, rs11549465 provides a functionally plausible candidate for testing whether inter-individual variation in the hypoxia-response pathway is associated with cancer susceptibility or progression. Previous studies have investigated rs11549465 in several cancer settings, although reported associations have varied according to tumor type, population, and environmental context [37,38,39]. The functional relevance of Pro582Ser substitution, including reports of altered HIF-1α transactivation, is also consistent with the broader literature on this variant [37,38,39].

This functional rationale is particularly relevant to the present study because smoking and excess body weight can contribute to oxidative, inflammatory, metabolic, and hypoxia-related stress. We therefore considered rs11549465 not simply as a candidate susceptibility marker, but as a variant whose association with melanoma might differ according to lifestyle-related exposures. Such combined analyses are intended to generate hypotheses and do not by themselves establish biological gene–environment interaction.

To our knowledge, the rs11549465 polymorphism has not previously been specifically investigated in cutaneous melanoma. Its evaluation in relation to both melanoma susceptibility and clinicopathological characteristics may therefore provide preliminary evidence as to whether this functionally relevant *HIF1A* variant warrants further investigation in melanoma.

We investigated the association of the non-synonymous *HIF1A* rs11549465 (1772 C>T; Pro582Ser) polymorphism, alone and in combination with overweight/obesity and smoking habits, with cutaneous melanoma susceptibility and clinicopathological characteristics in a Caucasian Italian cohort from Northeastern Italy.

## 2. Materials and Methods

### 2.1. Participants and Study Design

Patients with cutaneous melanoma and healthy controls were recruited at the Dermatology Clinic of Udine University Hospital [15,20,21], where routine diagnostic protocols were used to establish clinical status. The Institutional Ethics Committee of the University of Udine—“Azienda Sanitaria Universitaria Integrata di Udine”—approved the study protocol, which was conducted in accordance with the Declaration of Helsinki. The study had an observational case–control design. All participants provided written informed consent.

The study included 132 unrelated inpatients or outpatients of both sexes, aged 30–90 years, with documented diagnosis of cutaneous melanoma, and 312 asymptomatic healthy controls of both sexes in the same age range. Controls were recruited among healthy subjects attending the Dermatology Clinic for check-up visits and through announcements at the University Hospital. All participants resided in the Friuli-Venezia Giulia (FVG) region, located in Northeast Italy, within approximately 100 km of the city of Udine. Individual exposure to solar UV radiation, occupational sunlight, and specific environmental pollutants was not quantitatively measured, but we evaluated indoor tanning and sunburns. No participant lived or worked in areas of the neighboring Veneto region characterized by high per- and polyfluoroalkyl substance (PFAS) contamination [16]. This criterion was considered important in view of the association between melanoma and environmental pollution [7,16,40]. Inclusion criteria were self-reported Caucasian Italian ancestry and residence in the FVG region, located at the border with Austria and Slovenia, as previously described [13,14,15,20,21]. Genetic ancestry markers were not assessed. Exclusion criteria for healthy controls included any lifetime history of tumors and major acute or chronic diseases, such as severe autoimmune diseases, including type 1 diabetes [14,15,20,21].

Cutaneous melanoma was diagnosed by histopathological and immunohistochemical examination after excision of lesions with clinical and dermoscopic features suggestive of malignancy [15,20,21]. Disease stage was assigned from clinical, histological, and radiological findings, as previously described [20,21]. Only stage I-IV cutaneous melanomas were included; stage 0 in situ and mucosal melanomas were excluded. For patients with multiple melanomas, the index lesion was defined as the primary melanoma with the highest T category [20,21,41]. For clarity, only the six most prevalent histologic subtypes were specifically indicated [21]. Tumor-infiltrating lymphocytes (TILs) were classified as absent, non-brisk, or brisk according to Clark et al. [42]. Vascular emboli were defined as reported [13].

Non-metastatic melanoma (NMetM) was defined as stage I-II disease without metastasis after at least five years of follow-up from the initial melanoma diagnosis. Metastatic melanoma (MetM) comprised stage III-IV disease [15,20,21].

### 2.2. Anthropometric and Lifestyle Variables

Participants completed a structured questionnaire covering demographic characteristics, medical history, family history of skin cancers, smoking habits, and lifestyle, as previously described [15,20,21]. The phototype was classified according to Fitzpatrick criteria [43]. Smoking status was categorized as ever, current, or past smoking. Smoking intensity (≥10 and ≥20 cigarettes/day) and duration (≥10 and ≥20 years of smoking) were analyzed separately. Cumulative exposure was evaluated by pack-years, which were calculated as (cigarettes smoked per day/20) × years smoked; never-smokers were assigned zero. Pack-years were further categorized as ≥10 and ≥20 pack-years.

Body mass index (BMI) was calculated as weight in kilograms divided by height in meters squared (m^2^); 18.5 ≤ BMI < 25 kg/m^2^ indicated normal weight, 25 ≤ BMI < 30 kg/m^2^ indicated overweight, 30 ≤ BMI < 35 kg/m^2^ was considered obesity of class 1, and 35 ≤ BMI < 40 kg/m^2^ severe obesity of class 2; finally, BMI ≥ 25 kg/m^2^ indicated overweight/obesity [44].

Alcohol use was coded as a binary variable, and education was coded as a high-school diploma or university degree. These data were complete for all melanoma patients but incomplete for controls and were therefore only analyzed within the melanoma cohort.

### 2.3. Genetic Analysis of the HIF1A rs11549465 (1772 C>T) Polymorphism

The *HIF1A* rs11549465 polymorphism was determined by restriction-fragment analysis after PCR amplification of genomic DNA extracted from venous blood. PCR products were digested with the Bsl I restriction enzyme, as previously described [45]. Genotyping was repeated in 20% of samples with 100% concordance. Laboratory personnel were blinded to participants’ demographic and clinical data. Genotype distributions were assessed for Hardy–Weinberg equilibrium separately in melanoma patients and healthy controls as a quality-control check. Genotyping was successful in 444/444 participants (100% call rate).

### 2.4. Statistical Analysis

Continuous variables are presented as mean ± standard deviation (SD) and were compared using Student’s *t* test for independent samples. Hardy–Weinberg equilibrium was assessed separately in patients and controls using a chi-square test. For categorical comparisons, odds ratios (ORs), 95% confidence intervals (CIs), and two-sided Wald *p* values were calculated from 2 × 2 tables; a Haldane–Anscombe correction of 0.5 was applied when any cell was zero. Age- and sex-adjusted ORs for case–control comparisons were estimated by binary logistic regression. Additional exploratory models assessed BMI and smoking simultaneously: each model included one BMI threshold, one smoking measure, age, and sex; models comparing MetM with NMetM used age at melanoma diagnosis. A post hoc composite exposure was defined as BMI ≥ 25 kg/m^2^, ≥10 cigarettes/day, and ≥20 years of smoking. Allele-level comparisons were not adjusted because alleles within an individual are not independent. A sensitivity model comparing MetM with NMetM additionally included alcohol use and education together with smoking for ≥20 years, BMI ≥ 25 kg/m^2^, age at diagnosis, and sex.

All tests were two-sided. *p* ≤ 0.050 was considered statistically significant; 0.050 < *p* ≤ 0.100 is shown descriptively as a trend (denoted by ^). No correction for multiple comparisons was applied; therefore, subgroup, joint-exposure, and composite analyses were considered exploratory. Analyses were performed using SPSS for Windows, version 28, with independent computational verification of the tabulated and logistic regression results.

For the dominant genetic model (TT+CT vs. CC), statistical power was calculated using a two-sided α = 0.05, the observed 22.1% carrier frequency in controls, and the fixed sample sizes of 132 cases and 312 controls; the minimum detectable OR at 80% power was derived from the non-central chi-square distribution. The study had 80% power to detect ORs ≥ 1.88 or ≤0.42 under the dominant model, but only 41% power for an OR of 1.50.

## 3. Results

### 3.1. Overall Case–Control Comparisons

A total of 444 participants were genotyped for the rs11549465 polymorphism located in the *HIF1A* gene at position 1772. The mean age of 132 melanoma patients and 312 healthy controls was 60.7 ± 13.0 and 58.3 ± 19.4 years, respectively (*p* = 0.191). The proportion of men did not differ between melanoma patients and healthy controls (73/132 versus 163/312; OR = 1.20, CI = 0.80–1.80; *p* = 0.381). Genotype distributions conformed to Hardy–Weinberg equilibrium in melanoma patients (χ^2^ = 0.059, *p* = 0.809) and healthy controls (χ^2^ = 0.384, *p* = 0.536).

Table 1 summarizes genotype, allele, BMI, and smoking comparisons between melanoma patients and healthy controls. Genotype and allele frequencies did not differ between groups. After adjustment for age and sex, melanoma was associated with BMI ≥ 25 kg/m^2^ (adjusted OR [aOR] = 2.14, *p* = 0.001), CC plus BMI ≥ 25 kg/m^2^ (aOR = 2.13, *p* < 0.001), and CC plus BMI ≥ 30 kg/m^2^ (aOR = 2.08, *p* = 0.031); BMI ≥ 30 kg/m^2^ alone showed a trend (aOR = 1.75, *p* = 0.066 ^). Current smoking and CC plus current smoking were less frequent among melanoma patients (aOR = 0.42, *p* = 0.007 and aOR = 0.39, *p* = 0.019), whereas past smoking and CC plus past smoking were more frequent (aOR = 2.45, *p* < 0.001 and aOR = 2.17, *p* = 0.002). Smoking ≥ 10 or ≥20 cigarettes/day remained associated after adjustment (aOR = 1.85, *p* = 0.008 and aOR = 2.34, *p* = 0.004, respectively), as did smoking for ≥20 years (aOR = 2.34, *p* < 0.001). CC plus ≥ 10 cigarettes/day showed a trend (aOR = 1.52, *p* = 0.091 ^), whereas CC plus ≥ 20 cigarettes/day (aOR = 1.92, *p* = 0.049) and CC plus ≥ 20 years of smoking (aOR = 2.29, *p* = 0.003) were associated with melanoma. Mean pack-years were higher in melanoma patients than controls (9.4 ± 16.7 vs. 5.1 ± 12.2; *p* = 0.002); ≥10 and ≥20 pack-years remained associated after age- and sex-adjustment (aOR = 2.19, *p* = 0.001 and aOR = 3.31, *p* < 0.001, respectively).

In the present cohort, four melanoma patients and six controls had class 2 obesity. No participant had BMI ≥ 40 kg/m^2^. Four melanoma patients and eight controls were underweight (BMI < 18.5 kg/m^2^), but no participant had BMI < 18 kg/m^2^.

### 3.2. Metastatic Versus Non-Metastatic Melanoma

Table 2 compares 65 patients with metastatic melanoma (MetM; stages III–IV) and 67 patients with non-metastatic melanoma (NMetM; stages I–II). Genotype and allele frequencies did not differ. Smoking for ≥10 years (OR = 2.26, *p* = 0.024) or ≥20 years (OR = 3.35, *p* = 0.002) was more frequent in MetM than NMetM. CC plus ≥ 20 years of smoking was also associated with MetM (OR = 2.43, *p* = 0.036), whereas CC plus ≥ 10 years showed a trend (OR = 1.99, *p* = 0.072 ^). BMI and cigarette-intensity measures did not differ. Importantly, in a model including BMI ≥ 25 kg/m^2^, age at diagnosis, and sex, smoking for ≥20 years remained associated with MetM (aOR = 3.12, CI = 1.39–6.99, *p* = 0.006), whereas BMI ≥ 25 kg/m^2^ did not (aOR = 1.23, CI = 0.56–2.70, *p* = 0.612).

Mean pack-years were higher in MetM than NMetM (13.1 ± 21.0 vs. 5.9 ± 10.0; *p* = 0.013), and ≥20 pack-years was associated with MetM (OR = 3.05, *p* = 0.017). Alcohol use did not differ (OR = 1.02, *p* = 0.958), whereas high-school/university education was less frequent in MetM (OR = 0.39, *p* = 0.010). The triple combination BMI ≥ 25 kg/m^2^ + ≥10 cigarettes/day + ≥20 years smoking was more frequent in MetM than NMetM (OR = 2.66, *p* = 0.030).

Among past smokers, the time since smoking cessation was 19.7 ± 13.2 years in MetM and 21.9 ± 11.3 years in NMetM (*p* = 0.532). Neither melanoma subgroup differed from healthy past smokers (19.8 ± 13.6 years; *p* = 0.978 and *p* = 0.504, respectively).

Compared with healthy controls, MetM was associated with elevated BMI, past smoking, greater cigarette intensity, and longer smoking duration (Table 2). When BMI ≥25 kg/m^2^ and smoking for ≥20 years were entered simultaneously with age and sex, both remained independently associated with MetM versus controls (BMI: aOR = 2.26, CI = 1.21–4.22, *p* = 0.010; smoking duration: aOR = 3.85, CI = 2.08–7.14, *p* < 0.001). In a separate intensity model, BMI ≥ 25 kg/m^2^ (aOR = 2.44, *p* = 0.005) and ≥20 cigarettes/day (aOR = 2.76, *p* = 0.005) also remained associated. The post hoc composite exposure including the triple condition (BMI ≥ 25 kg/m^2^, ≥10 cigarettes/day, and ≥20 years of smoking) occurred in 19/65 MetM, 9/67 NMetM, and 20/312 controls. It was also associated with MetM versus controls after adjustment for age and sex (aOR = 5.95, CI = 2.82–12.55, *p* < 0.001), but its adjusted association within the melanoma cohort was only a trend (aOR = 2.39, CI = 0.95–6.06, *p* = 0.065 ^).

### 3.3. Joint Genotype–BMI and Genotype–Smoking Analyses

Table 3, Table 4 and Table 5 present exploratory comparisons of melanoma patients by combinations of the rs11549465 CC genotype with BMI or smoking exposures.

Table 3 compares melanoma patients with the CC genotype plus BMI ≥ 25 kg/m^2^ (n = 67) or plus BMI ≥ 30 kg/m^2^ (n = 19) with the corresponding remaining patients. Both exposure-defined subgroups were older at first melanoma diagnosis and included a higher proportion of men.

Genotype/allele and BMI variables that formed part of the subgroup definitions are reported in Supplementary Table S1. As expected, they produced very large ORs and were not interpreted as independent biological associations.

Among non-defining variables, smoking duration and intensity were more frequent in the CC plus BMI-defined subgroups.

Furthermore, CC plus BMI ≥ 25 kg/m^2^ was associated with an epithelioid cytological variant (OR = 2.92, *p* = 0.010) and vascular emboli (OR = 4.51, *p* = 0.025). CC plus BMI ≥ 30 kg/m^2^ was associated with stage III disease (OR = 4.35, *p* = 0.004), and head/neck melanoma (OR = 6.99, *p* = 0.002); TIL absence showed a trend (OR = 2.48, *p* = 0.071 ^).

Table 4 compares patients with the CC genotype plus ≥ 10 or ≥20 cigarettes/day. Genotype/allele and smoking related variables are shown in Supplementary Table S2. Beyond the expected associations with smoking variables (shown in Supplementary Table S2), both subgroups included more men, more patients with overweight/obesity, and alcohol drinkers. CC plus ≥ 10 cigarettes/day was associated with a lower frequency of non-brisk TILs (OR = 0.37, *p* = 0.034). It was also associated with a lower frequency of trunk melanoma (OR = 0.37, *p* = 0.012) and a higher frequency of hand/foot melanoma (OR = 5.00, *p* = 0.034) and head/neck melanoma (OR = 5.20, *p* = 0.007). CC plus ≥ 20 cigarettes/day was associated with head/neck melanoma (OR = 4.69, *p* = 0.015), TIL absence (OR = 5.48, *p* = 0.001) and a spindle-cell cytological variant (OR = 4.69, *p* = 0.015).

Table 5 compares patients with the CC genotype plus ≥ 10 or ≥20 years of smoking. Genotype/allele and smoking related variables are shown in Supplementary Table S3. Both exposure-defined subgroups were more frequently males, overweight and/or obese, and alcohol drinkers. The CC plus ≥ 10 years of smoking subgroup showed a trend toward metastatic disease (OR = 1.99, *p* = 0.072 ^) and was associated with a lower frequency of trunk melanoma (OR = 0.47, *p* = 0.046), a higher frequency of head/neck melanoma (OR = 4.17, *p* = 0.018), TIL absence (OR = 2.16, *p* = 0.048), and an epithelioid cytological variant (OR = 2.27, *p* = 0.044). Patients with the CC genotype plus ≥ 20 years of smoking had higher smoking intensity (Table S3). This subgroup was associated with metastatic melanoma (OR = 2.43, *p* = 0.036), stage III disease (OR = 2.74, *p* = 0.022), and head/neck melanoma (OR = 4.39, *p* = 0.014), and had a lower frequency of non-brisk TILs (OR = 0.36, *p* = 0.040).

Because several variables in Tables S1–S3 are components of the exposure-group definitions, the corresponding very large ORs are structural and should not be interpreted as independent biological associations.

### 3.4. Head and Neck Melanoma Analyses

Table 6 compares 13 patients with head/neck melanoma and 119 patients with melanoma at other sites. All head/neck melanoma patients (13/13, 100%) were CC carriers, and none were present smokers. Relative detailed data are shown in Supplementary Table S4. Head/neck melanoma was diagnosed at an older age (64.3 ± 10.1 versus 52.7 ± 13.7 years; *p* = 0.003), and none of these patients were diagnosed before age 50 (OR = 0.05, *p* = 0.043). Among melanoma patients, head/neck localization was associated with BMI ≥30 kg/m^2^ (OR = 5.14, *p* = 0.008), CC plus BMI ≥ 30 kg/m^2^ (OR = 6.99, *p* = 0.002), CC plus ≥ 10 cigarettes/day (OR = 5.20, *p* = 0.007), CC plus ≥ 20 cigarettes/day (OR = 4.69, *p* = 0.015), CC plus ≥ 10 years of smoking (OR = 4.17, *p* = 0.018), CC plus ≥ 20 years of smoking (OR = 4.39, *p* = 0.014), ≥10 pack-years (OR = 3.69, *p* = 0.031), CC plus ≥ 10 pack-years (OR = 6.02, *p* = 0.003), and ulceration (OR = 4.12, *p* = 0.025). Head/neck melanoma patients showed a trend for a higher frequency of stage IV disease (OR = 3.07, *p* = 0.061 ^).

By comparison of groups in Table 3, Table 4, Table 5 and Table 6, no statistically significant differences were observed across the corresponding comparisons for some histological subtypes, Breslow thickness, mitosis, regression, brisk positive TILs, or microsatellitosis (data are shown in Supplementary Tables S1–S4, respectively).

An additional exploratory comparison of the 13 patients with head/neck melanoma and the 312 healthy controls showed, after adjustment for age and sex, significant associations with CC plus BMI ≥ 25 kg/m^2^, BMI ≥ 30 kg/m^2^, and CC plus BMI ≥ 30 kg/m^2^, past smoking, CC plus past smoking, ≥10 cigarettes/day, CC plus ≥ 10 cigarettes/day, ≥20 cigarettes/day, CC plus ≥ 20 cigarettes/day, CC plus ≥ 10 years of smoking, ≥20 years of smoking, and CC plus ≥ 20 years of smoking, ≥10 pack-years, CC plus ≥ 10 pack-years, and CC plus ≥ 20 pack-years. These estimates are exploratory, are based on a small subgroup, and have wide confidence intervals.

Finally, none of the following four variables differed significantly in any comparison reported in Table 3, Table 4, Table 5 and Table 6: multiple melanomas, additional non-melanoma skin cancer, additional non-skin cancer, or death within five years of the first melanoma diagnosis.

## 4. Discussion

In this observational case–control study, we investigated the association between the *HIF1A* rs11549465 (1772 C>T) polymorphism and cutaneous melanoma in a Caucasian Italian population from Northeastern Italy. Among healthy controls, genotype frequencies were TT 1.3%, CT 20.8%, and CC 77.9%; in the overall cohort, the corresponding frequencies were 1.4%, 21.6%, and 77.0%. These distributions were broadly similar to those reported in 212 Slovenian patients with malignant mesothelioma (TT 8.0%, CT 18.9%, and CC 73.1%) [46] and in Austrian healthy controls (TT+CT 17.8% and CC 82.2%) [47]. Slovenia and Austria border the Italian FVG region, where participants are recruited and may share partially similar geographical and genetic backgrounds. In a meta-analysis including 8633 Asian and Caucasian controls, genotype frequencies were TT 0.6%, CT 12.7%, and CC 86.6% [37]. Globally, allele frequencies were C 92.8% and T 7.2%, whereas European populations showed C 90.0% and T 10.0% [48].

Overall, in our study, the distribution of rs11549465 genotypes and alleles did not differ significantly between patients with cutaneous melanoma and healthy controls, suggesting that this polymorphism is not independently associated with melanoma susceptibility in the present cohort.

Exploratory analyses nevertheless identified associations between metabolic or smoking exposures and selected melanoma characteristics. The strongest within-patient findings involved smoking duration, metastatic status, and head/neck localization. Because subgroup sizes were small and many variables were correlated or embedded in the subgroup definitions, these results should be interpreted as hypothesis-generating joint associations rather than evidence of gene–environment interaction; formal interaction terms were not tested.

Hypoxia and oxidative stress are key components of the tumor microenvironment and are increasingly recognized as relevant mechanisms in melanoma biology [3,17,27,35,36]. HIF-1α is a central transcriptional regulator of cellular adaptation to low oxygen availability and controls several pathways involved in angiogenesis (mainly through VEGF activation), metabolic reprogramming, cell survival, invasion, and immune modulation [27]. In melanoma, HIF-1α expression has been associated with melanoma aggressiveness, metastatic potential, and radiotherapy resistance [35,36,49]. The rs11549465 polymorphism, corresponding to the 1772 C>T variation and the Pro582Ser amino acid substitution, has been investigated in several cancers because of its potential functional effect on HIF-1α transactivation activity, and the T allele has been associated with poor prognosis [37,38,39]. Nevertheless, to our knowledge, this is the first study evaluating this polymorphism in relation to cutaneous melanoma. The reported increase in transactivation by Pro582Ser substitution could affect VEGF-mediated angiogenesis, glycolysis, immune evasion, and oxidative-stress responses; however, these mechanisms were not tested here.

The lack of a significant difference in rs11549465 genotype and allele frequencies between melanoma patients and controls indicates that this SNP does not appear to represent a major independent susceptibility marker for cutaneous melanoma in this population. This finding is relevant because previous meta-analyses on different cancer types have suggested an association between rs11549465 and overall cancer risk, although findings vary according to tumor type, ancestry, and study design [37,38,39,45,46,47,50]. Most investigations associated the T variant with increased cancer risk [37,38,39]; however, a meta-analysis suggested that the C variant of HIF-1α C1772T polymorphism may increase the risk of gastrointestinal tract cancer, especially in Asian populations [51].

Melanoma has a complex multifactorial etiology, in which ultraviolet radiation exposure, pigmentary phenotype, nevus burden, immune regulation, family history, and somatic driver mutations play prominent roles [2,3,6]. In this context, the contribution of a single germline polymorphism may be modest and difficult to detect, particularly in relatively small cohorts.

In the present case–control analysis, overweight/obesity was approximately twofold more frequent among melanoma patients, and the associations for BMI ≥ 25 kg/m^2^ and CC plus BMI ≥ 25 kg/m^2^ remained significant after adjustment for age and sex. CC plus BMI ≥ 30 kg/m^2^ also remained associated, whereas BMI ≥ 30 kg/m^2^ alone showed a trend. In mutually adjusted models comparing MetM with controls, BMI ≥ 25 kg/m^2^ remained associated after accounting for smoking duration or intensity. By contrast, within the melanoma cohort, BMI did not remain associated with metastatic status after adjustment, whereas smoking for ≥20 years and ≥20 pack-years did. BMI was assessed at a single time point, often after first melanoma diagnosis; therefore, temporality and causality cannot be inferred. Obesity is associated with chronic inflammation, altered adipokine secretion, insulin resistance, and oxidative stress [26,52]. These conditions may influence melanoma biology and treatment response, although the relevance of BMI to melanoma risk and prognosis remains debated [52,53,54]. A recent meta-analysis associated overweight/obesity with increased risks of several cancers, with heterogeneity determined according to geographical region. Higher BMI was associated with increased risks of head and neck cancers, whereas findings for melanoma were not significant [55].

The observed associations involving the CC genotype and overweight/obesity provide a rationale for considering metabolic and inflammatory pathways together with HIF-1α biology. Reduced tissue perfusion and adipose-tissue hypoxia can activate HIF and NF-κB pathways in obesity [56,57]. HIF-1 has roles in insulin resistance, and rs11549465 polymorphism has been reported to influence nuclear factor kappa B subunit 1 (NF-κB1) transcription factor, which in turn, modulates over 200 target genes involved in immune function and obesity [58]. The *HIF1A* rs11549465 polymorphism has also been associated with cellulite [59,60]. However, the present data do not demonstrate that *HIF1A* rs11549465 polymorphism mediates the effect of obesity on melanoma.

In our study, the clearest within-patient signal concerned smoking duration. Smoking for ≥20 years remained associated with metastatic melanoma after adjustment for BMI, age at diagnosis, and sex. Consistently, cumulative smoking exposure expressed as pack-years—a measure associated with the risk of several smoking-related cancers [61]—was higher in MetM than NMetM patients (13.1 ± 21.0 versus 5.9 ± 10.0 pack-years; *p* = 0.013), and ≥20 pack-years was associated with MetM (OR = 3.05, CI = 1.22–7.58; *p* = 0.017). The CC plus ≥ 20 years of smoking subgroup was also associated with metastatic melanoma (OR = 2.43) and stage III disease (OR = 2.74), whereas CC plus ≥ 20 cigarettes/day was associated with TIL absence (OR = 5.48). The post hoc three-exposure composite (BMI ≥ 25 kg/m^2^, ≥10 cigarettes/day, and ≥20 years of smoking) was sufficiently frequent for analysis, but its adjusted within-patient association weakened to a trend. These findings require replication and should not be interpreted mechanistically.

Smoking is a major source of reactive oxygen species and can impair immune surveillance, modify inflammatory responses, and promote DNA damage [61,62]. In tumors, chronic exposure to smoking-related oxidative stress may reinforce hypoxia-driven signaling pathways and favor invasive behavior. Because HIF-1α is strongly linked to cellular responses to hypoxia and oxidative stress, the observed joint associations provide biological context for examining genotype together with smoking, although they do not demonstrate a gene–environment interaction. However, this scenario is consistent with the possibility that the CC genotype alone does not increase melanoma risk, whereas prolonged smoking-related stress could modify its biological relevance and potentially contribute to conditions associated with metastatic progression. Studies in cancer cells reported lower transcriptional activity of the *HIF1A* rs11549465 C allele than the rare T variant [60]. On this basis, one possible, but unproven, interpretation is that the CC genotype may be associated with a reduced HIF-1α response to smoking-related cellular stress. This interpretation is indirectly compatible with studies of benzene, a carcinogen present in cigarette smoke, in which higher HIF-1α expression was associated with partial protection against cellular damage [63]. However, these findings derive from distinct experimental contexts, and the present epidemiological data establish neither a mechanism nor a genotype–smoking interaction. Collectively, these observations provide mechanistic hypotheses but cannot explain the present epidemiological associations without functional validation.

HIF activation is regulated not only by changes in oxygen partial pressure but also by other external factors, including nitric oxide, pro-inflammatory cytokines such as IL-1β, and tobacco-derived molecules [25,64,65,66]. One study showed that cigarette-smoke extract increased reactive oxygen species levels and stabilized HIF-1α in primary human cells. It also upregulated a hypoxia-induced gene set, including *NFKB1*, even under normoxic conditions [66]. These findings support the biological plausibility of a role for HIF-1α in cigarette-smoke-induced cellular stress and inflammation [25,65,66], but they do not establish causality in the present study.

Cigarette smoke contains numerous carcinogens, including nitrosamines, aldehydes, benzene, benzo[a]pyrene, metals, and radioactive isotopes [61,62]. Among these, arsenic induces oxidative stress and has been associated with melanoma [40], whereas polonium-210 (^210^Po) and lead-210 (^210^Pb) may enter the circulation after smoking inhalation and accumulate in tissues, including bone [67,68]. Smoking 20 cigarettes per day has been estimated to result in an annual effective radiation dose of approximately 0.3 mSv [67]. In smokers, ^210^Po has been detected in blood, urine, and hair, with urinary levels correlating with age, smoking intensity, and duration [69]. Urinary levels were comparable between smokers of <10 cigarettes per day and non-smokers but increased markedly in those smoking ≥ 20 cigarettes per day [67,69].

In our cohort, most past smokers had stopped smoking approximately 20 years before enrolment, suggesting possible long-lasting effects of smoking due to the accumulation of toxic substances and/or epigenetic changes [14]. However, recall bias, changes in smoking after diagnosis, and residual confounding are alternative explanations.

A recent meta-analysis found that ever-smokers had a higher risk of complications from sentinel-node biopsy and lymph node dissection than never-smokers, and that melanoma-specific mortality was higher in current than never-smokers [70].

In our cohort, melanoma patients were less frequently current smokers than healthy controls. This may partly reflect advice to stop smoking after the detection or diagnosis of suspicious or malignant skin lesions. The association of prolonged smoking with metastatic disease supports routine smoking-cessation counseling, but not *HIF1A* genotyping or melanoma-specific risk stratification.

Associations with head/neck localization were also notable. Compared with melanomas at other sites, head/neck melanomas were associated with CC plus BMI ≥ 30 kg/m^2^ (OR = 6.99), CC plus ≥ 10 cigarettes/day (OR = 5.20), CC plus ≥ 20 cigarettes/day (OR = 4.69), CC plus ≥ 10 years of smoking (OR = 4.17), and CC plus ≥ 20 years of smoking (OR = 4.39). Patients with head/neck melanoma were older at diagnosis, and no patient was diagnosed before age 50. These findings are consistent with the older age reported in other cohorts [71,72,73,74,75,76]. However, the wide confidence intervals emphasize the limited precision of these estimates based on only 13 head/neck cases. Interestingly, in our study, head/neck melanoma patients did not differ in phototype, nevi number, sunburns or indoor tanning from melanoma localized in other sites. Compared with healthy controls, adjusted associations were observed for selected BMI- and smoking-related variables, with aORs ranging from 3.41 to 9.07, although wide confidence intervals and correlated exposures make the findings hypothesis-generating.

Head and neck skin is chronically exposed to ultraviolet radiation and may accumulate both UV-induced damage and environmental oxidative insults. Interestingly, HIF-1α is highly expressed in head and neck squamous cell carcinoma [50]; however, this malignancy is biologically distinct from melanoma. High-intensity or prolonged smoking, together with a genotype potentially involved in hypoxia-response regulation, could nevertheless contribute to increased oxidative stress, altered vascular or lymphatic responses, and impaired antitumor immunity. UV-related mechanisms in cutaneous melanoma remain incompletely understood [71,72,73], and evidence linking sun exposure to head/neck melanoma localization is inconsistent, possibly reflecting site-specific etiological mechanisms [74,75,76]. A Greek study found fewer nevi in patients with head/neck or lower-extremity melanoma than in those with trunk melanoma [77]. Consistently, in our study, ≥50 nevi showed a trend toward a lower frequency in head/neck than in other-site melanoma (OR = 0.27, *p* = 0.053 ^).

Anatomical localization is clinically relevant and is included in nomograms estimating melanoma recurrence, survival, and sentinel lymph node positivity [78,79]. Immune-related genetic variants may also influence localization: the *IL1B* rs1143634 polymorphism was associated with upper-limb melanoma [20], whereas *IL1B* rs16944 was associated with lower-limb melanoma [21]. IL-1β and HIF-1α participate in a positive feedback loop involving HIF-1α upregulation, IL-1β production, and inflammasome activation [80,81,82]. In cancer, this axis links inflammation and hypoxia with angiogenesis and metastasis [27,30,80,81]. These observations raise, but do not establish, the possibility that genetic variation affecting immunity and hypoxia/oxidative stress responses contributes to melanoma localization, potentially partly independently of UV exposure.

The association between the CC genotype combined with ≥20 cigarettes/day and TIL absence may be relevant because TILs reflect the host antitumor immune response. Of note, adoptive TIL therapy is used in selected patients with advanced melanoma, although histological TIL absence is not itself a treatment indication [6,83]. HIF-1α regulates the tumor immune microenvironment, whereas smoking can impair immune function and promote inflammation. However, the present data do not establish these mechanisms, and this subgroup finding is based on small numbers and requires independent confirmation.

### 4.1. Strengths and Limitations

Several limitations should be considered. First, the sample size, particularly after stratification by metastatic status, genotype, BMI, and smoking variables, was limited. This may have reduced statistical power and increased the risk of unstable estimates in subgroup analyses. Second, the observational case–control design does not allow causal inference. Third, lifestyle variables such as smoking history and body weight were based on collected clinical and questionnaire data and may be subject to recall or classification bias, and passive smoking was not assessed. Fourth, the study population consisted of Caucasian Italian individuals from a geographically defined area; therefore, the findings may not be generalizable to other ethnic groups or populations with different environmental exposures. Fifth, no correction for multiple comparisons was applied. Finally, no functional assays or formal interaction tests were performed; therefore, the biological mechanisms underlying the observed associations remain hypothetical. The power analysis only demonstrated adequate power for relatively large dominant-model effects, not modest effects, the rare TT genotype, or small subgroups. Alcohol and education data were incomplete in controls; individual UV exposure, occupational sun exposure, income, and other socioeconomic measures were not available for adjustment. Residual confounding remains possible, and results may not generalize to populations with different ancestries, allele frequencies, exposures, or melanoma incidence.

Despite these limitations, the present study has several strengths: it addresses a previously unexplored association between *HIF1A* rs11549465 and cutaneous melanoma, includes a well-defined case–control cohort, and considers both genetic and lifestyle-related variables. The joint analyses generate hypotheses about metabolic, smoking, and *HIF1A*-related pathways in melanoma. They do not support clinical genotyping or therapeutic decisions but may inform larger prospective studies and functional work. Our study may contribute to the debate and innovative research regarding the possible use of HIF-1α inhibitors to block growth and vascularization of melanoma, head/neck and other tumors, and also to overcome resistance to radiotherapy and anti-PD-1 immunotherapy [28,49,84].

### 4.2. Novelty, Clinical Significance, and Future Directions

To our knowledge, this is the first study to evaluate *HIF1A* rs11549465 in cutaneous melanoma, either alone or together with BMI and detailed smoking measures. The polymorphism alone was not associated with melanoma susceptibility. Prolonged smoking, BMI, and selected joint CC-genotype/lifestyle exposures showed associations with metastatic or head/neck melanoma. These subgroup findings are exploratory and do not currently support clinical genotyping or risk prediction, but they provide hypotheses for validation in larger, independent cohorts.

The principal potentially actionable observation concerns prolonged smoking rather than the *HIF1A* genotype. Because smoking is a modifiable exposure, confirmation of its association with melanoma progression would reinforce smoking-cessation counseling and could justify incorporation of detailed cumulative tobacco history into prospective melanoma prognostic studies. At present, however, these data should not alter melanoma staging, surveillance schedules, or treatment selection.

In the continuing debate on overweight/obesity, smoking, and cutaneous melanoma, our study provides additional observational evidence consistent with previous findings in an Italian cohort [14]. BMI ≥ 25 kg/m^2^ and smoking for ≥20 years and ≥20 pack-years were independently associated with MetM versus controls after adjustment; among melanoma patients, prolonged smoking was also associated with metastatic disease. The composite exposure comprising BMI ≥ 25 kg/m^2^, ≥10 cigarettes/day, and ≥20 years of smoking showed a stronger association with MetM versus controls (aOR = 5.95, *p* < 0.001). These observational findings do not establish causality but may inform further research and prevention strategies [8,9,78,85]. Because modifiable factors such as smoking, unhealthy diet, and obesity contribute substantially to the overall cancer burden [8,9,55], their potential role in melanoma risk and progression warrants further evaluation within precision-prevention approaches [6,8,9,78,85]. The association of prolonged smoking with metastatic melanoma supports routine smoking-cessation counseling.

Future studies should include larger multicenter cohorts, prespecified hypotheses, external validation, formal genotype-by-exposure interaction models, and more complete adjustment for UV-related and other melanoma risk factors. A prospective assessment of smoking should ideally include longitudinal changes after diagnosis. Functional studies should determine whether rs11549465 is associated with HIF-1α protein abundance or transcriptional activity in melanoma tissue and whether such effects vary according to tobacco exposure or adiposity.

Integrating germline *HIF1A* variation with tumor characteristics—including *BRAF*, *NRAS*, and *TERT* alterations, PD-L1 expression and other immune markers—as well as transcriptomic profiles, circulating biomarkers, and direct measures of hypoxia or oxidative stress could help determine whether the epidemiological associations observed here correspond to reproducible biological phenotypes. Such studies could also evaluate whether *HIF1A* variation is related to treatment response or resistance, but the present data provide no basis for therapeutic stratification according to rs11549465 genotype.

## 5. Conclusions

The *HIF1A* rs11549465 polymorphism was not independently associated with cutaneous melanoma susceptibility in this Italian cohort. Prolonged smoking remained associated with metastatic status after adjustment for BMI, age at diagnosis, and sex, whereas BMI did not distinguish metastatic from non-metastatic patients. Compared with healthy controls, both BMI ≥ 25 kg/m^2^ and prolonged smoking remained associated with metastatic melanoma in mutually adjusted models. Interestingly, joint CC-genotype/lifestyle associations were observed for head/neck localization. However, because subgroup sizes were small, exposures were correlated, and multiple comparisons were performed without correction, these results should be considered hypothesis-generating.

Larger prospective cohorts, prespecified interaction models, and functional studies are required to determine whether these observations reflect reproducible biological interactions.

Overall, the primary genetic finding of this study is the absence of a detectable independent association between the rs11549465 polymorphism and melanoma susceptibility. The more consistent observational signal concerned prolonged smoking, which warrants further investigation as potentially relevant modifiable exposure in melanoma progression.

The study may contribute to the ongoing search for biomarkers that improve prognostic tools and clinical management of melanoma, ultimately enhancing patient’s outcomes [78,85]. Taken together, these findings suggest that *HIF1A*-related hypoxia-response variation may have context-dependent relevance to advanced-stage disease and anatomical localization. Although exploratory, this hypothesis may inform studies of HIF-1α-related pathways in cancer biology and immunomodulation, and possible HIF-1α-targeted therapies. It will also be important to assess whether *HIF1A* polymorphisms or linked variants contribute to inter-individual variability in melanoma immunotherapy response [46,50,60].

Functional studies are required to determine the biological relevance of *HIF1A* variants alone or jointly with lifestyle exposures in cancer development, progression, and localization. Future studies should evaluate this variant in larger multicenter cohorts with prespecified interaction analyses and functional integration of germline *HIF1A* variation with BRAF, NRAS, TERT, PD-L1, transcriptomic, and circulating-biomarker data.

Until such evidence is available, the present findings do not support rs11549465 genotyping for melanoma risk prediction, prognostic stratification, or therapeutic decision-making.

## Figures and Tables

**Table 1:** Genotype and allele frequencies of *HIF1A* rs11549465 (1772 C>T), BMI, and smoking variables in 132 cutaneous melanoma patients and 312 healthy controls.

Variable	Melanoma Patients (n = 132)	Healthy Controls (n = 312)	Unadjusted OR (CI), *p*	Age- and Sex-Adjusted OR (CI), *p*
***HIF1A* genotype**				
TT, n (%)	2 (1.5%)	4 (1.3%)	1.18 (0.21–6.55), 0.846	1.13 (0.20–6.27), 0.890
CT, n (%)	31 (23.5%)	65 (20.8%)	1.17 (0.72–1.90), 0.535	1.16 (0.71–1.90), 0.542
CC, n (%)	99 (75.0%)	243 (77.9%)	0.85 (0.53–1.37), 0.509	0.86 (0.53–1.38), 0.525
TT+CT, n (%)	33 (25.0%)	69 (22.1%)	1.17 (0.73–1.89), 0.509	1.17 (0.72–1.88), 0.525
***HIF1A* allele**				
Allele T, n (%)	35/264 (13.3%)	73/624 (11.7%)	1.15 (0.75–1.78), 0.516	—
Allele C, n (%)	229/264 (86.7%)	551/624 (88.3%)	0.87 (0.56–1.33), 0.516	—
BMI ≥ 25 kg/m^2^, n (%)	85 (64.4%)	145 (46.5%)	**2.08 (1.37–3.17), <0.001**	**2.14 (1.36–3.37), 0.001**
CC + BMI ≥ 25 kg/m^2^, n (%)	67 (50.8%)	102 (32.7%)	**2.12 (1.40–3.21), <0.001**	**2.13 (1.37–3.32), <0.001**
BMI ≥ 30 kg/m^2^, n (%)	23 (17.4%)	32 (10.3%)	**1.85 (1.03–3.30), 0.038**	1.75 (0.96–3.16), 0.066 ^
CC + BMI ≥ 30 kg/m^2^, n (%)	19 (14.4%)	22 (7.1%)	**2.22 (1.16–4.25), 0.017**	**2.08 (1.07–4.05), 0.031**
Ever smoker, n (%)	64 (48.5%)	129 (41.3%)	1.34 (0.89–2.01), 0.166	1.27 (0.83–1.95), 0.276
CC + ever smoker, n (%)	48 (36.4%)	95 (30.4%)	1.31 (0.85–2.00), 0.223	1.24 (0.79–1.93), 0.350
Present smoker, n (%)	13 (9.8%)	65 (20.8%)	**0.42 (0.22–0.78), 0.007**	**0.42 (0.22–0.79), 0.007**
CC + present smoker, n (%)	8 (6.1%)	44 (14.1%)	**0.39 (0.18–0.86), 0.019**	**0.39 (0.18–0.86), 0.019**
Past smoker, n (%)	51 (38.6%)	64 (20.5%)	**2.44 (1.56–3.81), <0.001**	**2.45 (1.52–3.95), <0.001**
CC + past smoker, n (%)	40 (30.3%)	51 (16.3%)	**2.23 (1.38–3.59), 0.001**	**2.17 (1.32–3.58), 0.002**
Cigarettes/day ≥ 10 ever, n (%)	49 (37.1%)	74 (23.7%)	**1.90 (1.22–2.95), 0.004**	**1.85 (1.18–2.91), 0.008**
CC + cigarettes/day ≥ 10 ever, n (%)	36 (27.3%)	60 (19.2%)	1.58 (0.98–2.53), 0.061 ^	1.52 (0.94–2.47), 0.091 ^
Cigarettes/day ≥ 20 ever, n (%)	27 (20.5%)	30 (9.6%)	**2.42 (1.37–4.26), 0.002**	**2.34 (1.31–4.18), 0.004**
CC + cigarettes/day ≥ 20 ever, n (%)	19 (14.4%)	24 (7.7%)	**2.02 (1.06–3.83), 0.032**	**1.92 (1.00–3.68), 0.049**
≥10 years smoking ever, n (%)	54 (40.9%)	106 (34.0%)	1.35 (0.89–2.05), 0.165	1.28 (0.83–1.98), 0.266
CC + ≥10 years smoking ever, n (%)	41 (31.1%)	83 (26.6%)	1.24 (0.80–1.94), 0.339	1.18 (0.74–1.87), 0.482
≥20 years smoking ever, n (%)	42 (31.8%)	51 (16.3%)	**2.39 (1.49–3.84), <0.001**	**2.34 (1.42–3.83), <0.001**
CC + ≥20 years smoking ever, n (%)	32 (24.2%)	37 (11.9%)	**2.38 (1.41–4.02), 0.001**	**2.29 (1.33–3.95), 0.003**
Pack-years, mean ± SD	9.4 ± 16.7	5.1 ± 12.2	—, **0.002**	—
≥10 pack-years, n (%)	44 (33.3%)	57 (18.3%)	**2.24 (1.41–3.55), <0.001**	**2.19 (1.35–3.55), 0.001**
CC + ≥10 pack-years, n (%)	33 (25.0%)	46 (14.7%)	**1.93 (1.17–3.19), 0.011**	**1.85 (1.10–3.10), 0.020**
≥20 pack-years, n (%)	27 (20.5%)	22 (7.1%)	**3.39 (1.85–6.21), <0.001**	**3.31 (1.76–6.21), <0.001**
CC + ≥20 pack-years, n (%)	19 (14.4%)	18 (5.8%)	**2.75 (1.39–5.42), 0.004**	**2.59 (1.28–5.24), 0.008**

Unadjusted ORs and 95% CIs were calculated from 2 × 2 tables. Adjusted ORs were estimated by logistic regression including age and sex (complete-case analysis: 132 patients and 312 controls; n = 444). Allele-level comparisons were not adjusted. Statistically significant estimates are shown in bold; trends are denoted by ^. Continuous pack-years were compared using Student’s *t* test and were not entered in the adjusted table column. OR and CI were not calculable for continuous variables. Not calculable values were indicated by —.

**Table 2:** Comparisons of *HIF1A* rs11549465 (1772 C>T), BMI, and smoking variables among metastatic melanoma (MetM; n = 65), non-metastatic melanoma (NMetM; n = 67), and 312 healthy controls.

Variable	MetM (n = 65)	NMetM (n = 67)	MetM vs. NMetM OR (CI), *p*	MetM vs. Controls OR (CI), *p*	NMetM vs. Controls OR (CI), *p*
***HIF1A* genotype**					
TT, n %	2 (3.1%)	0 (—)	5.31 (0.25–112.9), 0.284	2.52 (0.45–14.03), 0.293	0.52 (0.03–9.82), 0.665
CT, n (%)	13 (20.0%)	18 (26.9%)	0.68 (0.30–1.53), 0.354	0.83 (0.43–1.62), 0.592	1.23 (0.67–2.23), 0.505
CC, n (%)	50 (76.9%)	49 (73.1%)	1.22 (0.56–2.70), 0.616	1.07 (0.57–2.01), 0.834	0.87 (0.48–1.59), 0.658
TT+CT, n (%)	15 (23.1%)	18 (26.9%)	0.82 (0.37–1.80), 0.616	0.93 (0.50–1.76), 0.834	1.14 (0.63–2.08), 0.658
***HIF1A* allele**					
Allele T, n (%)	17/130 (13.1%)	18/134 (13.4%)	0.97 (0.48–1.98), 0.932	1.03 (0.59–1.80), 0.925	1.06 (0.61–1.83), 0.836
Allele C, n (%)	113/130 (86.9%)	116/134 (86.6%)	1.03 (0.51–2.10), 0.932	0.97 (0.56–1.70), 0.925	0.94 (0.55–1.63), 0.836
BMI ≥ 25 kg/m^2^, n (%)	45 (69.2%)	40 (59.7%)	1.52 (0.74–3.11), 0.254	**2.73 (1.54–4.83), <0.001**	**1.80 (1.05–3.07), 0.032**
CC + BMI ≥ 25 kg/m^2^, n (%)	37 (56.9%)	30 (44.8%)	1.63 (0.82–3.24), 0.164	**2.84 (1.65–4.89), <0.001**	**1.74 (1.02–2.97), 0.043**
BMI ≥ 30 kg/m^2^, n (%)	14 (21.5%)	9 (13.4%)	1.77 (0.71–4.43), 0.223	**2.48 (1.24–4.97), 0.010**	1.40 (0.64–3.09), 0.403
CC + BMI ≥ 30 kg/m^2^, n (%)	12 (18.5%)	7 (10.4%)	1.94 (0.71–5.29), 0.195	**3.08 (1.44–6.59), 0.004**	1.59 (0.65–3.88), 0.313
Ever smoker, n (%)	35 (53.8%)	29 (43.3%)	1.53 (0.77–3.04), 0.226	**1.74 (1.02–2.97), 0.044**	1.14 (0.67–1.93), 0.639
CC + ever smoker, n (%)	27 (41.5%)	21 (31.3%)	1.56 (0.76–3.18), 0.225	1.69 (0.98–2.93), 0.061 ^	1.09 (0.61–1.92), 0.776
Present smoker, n (%)	8 (12.3%)	5 (7.5%)	1.74 (0.54–5.63), 0.355	0.55 (0.25–1.22), 0.141	**0.32 (0.12–0.82), 0.018**
CC + present smoker, n (%)	4 (6.2%)	4 (6.0%)	1.03 (0.25–4.32), 0.965	0.41 (0.14–1.19), 0.102	0.40 (0.14–1.15), 0.090 ^
Past smoker, n (%)	27 (41.5%)	24 (35.8%)	1.27 (0.63–2.57), 0.500	**2.85 (1.62–5.02), <0.001**	**2.24 (1.27–3.96), 0.005**
CC + past smoker, n (%)	23 (35.4%)	17 (25.4%)	1.61 (0.76–3.41), 0.212	**2.90 (1.61–5.23), <0.001**	1.80 (0.96–3.37), 0.066 ^
Cigarettes/day ≥ 10 ever, n (%)	27 (41.5%)	22 (32.8%)	1.45 (0.71–2.95), 0.302	**2.37 (1.36–4.14), 0.002**	1.63 (0.92–2.89), 0.093 ^
CC + cigarettes/day ≥ 10 ever, n (%)	20 (30.8%)	16 (23.9%)	1.42 (0.66–3.06), 0.375	**1.93 (1.06–3.51), 0.030**	1.36 (0.73–2.56), 0.332
Cigarettes/day ≥ 20 ever, n (%)	16 (24.6%)	11 (16.4%)	1.66 (0.70–3.92), 0.246	**3.17 (1.61–6.24), <0.001**	1.91 (0.90–4.02), 0.091 ^
CC + cigarettes/day ≥ 20 ever, n (%)	12 (18.5%)	7 (10.4%)	1.94 (0.71–5.29), 0.195	**2.80 (1.32–5.94), 0.007**	1.44 (0.59–3.50), 0.417
≥10 years smoking ever, n (%)	33 (50.8%)	21 (31.3%)	**2.26 (1.11–4.59), 0.024**	**2.09 (1.22–3.59), 0.007**	0.93 (0.53–1.63), 0.790
CC + ≥10 years smoking ever, n (%)	25 (38.5%)	16 (23.9%)	1.99 (0.94–4.22), 0.072 ^	**1.79 (1.03–3.13), 0.041**	0.90 (0.49–1.66), 0.736
≥20 years smoking ever, n (%)	29 (44.6%)	13 (19.4%)	**3.35 (1.54–7.29), 0.002**	**4.26 (2.40–7.57), <0.001**	1.27 (0.65–2.50), 0.482
CC+ ≥20 years smoking ever, n (%)	21 (32.3%)	11 (16.4%)	**2.43 (1.06–5.57), 0.036**	**3.66 (1.97–6.83), <0.001**	1.51 (0.73–3.13), 0.271
Pack-years, mean ± SD	13.1 ± 21.0	5.9 ± 10.0	**—, 0.013**	**—, <0.001**	—, 0.586
≥10 pack-years, n (%)	26 (40.0%)	18 (26.9%)	1.81 (0.87–3.78), 0.111	**2.98 (1.68–5.29), <0.001**	1.64 (0.89–3.03), 0.111
CC + ≥10 pack-years, n (%)	19 (29.2%)	14 (20.9%)	1.56 (0.71–3.46), 0.271	**2.39 (1.29–4.44), 0.006**	1.53 (0.78–2.98), 0.213
≥20 pack-years, n (%)	19 (29.2%)	8 (11.9%)	**3.05 (1.22–7.58), 0.017**	**5.44 (2.74–10.84), <0.001**	1.79 (0.76–4.21), 0.184
CC + ≥20 pack-years, n (%)	13 (20.0%)	6 (9.0%)	2.54 (0.90–7.16), 0.078 ^	**4.08 (1.89–8.84), <0.001**	1.61 (0.61–4.21), 0.335
BMI ≥ 25 kg/m^2^ + ≥10 cigarettes/day + ≥20 years smoking, n (%)	19 (29.2%)	9 (13.4%)	**2.66 (1.10–6.43), 0.030**	**6.03 (2.99–12.15), <0.001**	2.27 (0.98–5.23), 0.055 ^
Alcohol drinker, n (%)	42 (64.6%)	43 (64.2%)	1.02 (0.50–2.08), 0.958	—	—
High-school diploma or university degree, n (%)	30 (46.2%)	46 (68.7%)	**0.39 (0.19–0.80), 0.010**	—	—

Differences between groups were evaluated using ORs and CIs for categorical variables. A Haldane–Anscombe correction was applied when a 2 × 2 table contained a zero cell. The three-exposure composite was defined post hoc as BMI ≥ 25 kg/m^2^, ≥10 cigarettes/day, and ≥20 years of smoking. Statistically significant estimates are shown in bold; trends are denoted by ^. Continuous pack-years were compared using Student’s *t* test, thus OR and CI were not calculable. Alcohol and education comparisons were restricted to MetM versus NMetM because control data were incomplete. OR and CI were not calculable for continuous variables. Not calculable values were indicated by —.

**Table 3:** Comparison of demographic and clinicopathological characteristics between melanoma patients with *HIF1A* rs11549465 CC plus BMI ≥ 25 kg/m^2^ (n = 67) and the remaining patients (n = 65), and between patients with CC plus BMI ≥ 30 kg/m^2^ (n = 19) and the remaining patients (n = 113).

Variable	CC + BMI ≥ 25 kg/m^2^ (n = 67)	Other Patients (n = 65)	OR (CI), *p*	CC + BMI ≥ 30 kg/m^2^ (n = 19)	Other Patients (n = 113)	OR (CI), *p*
Age < 50 years at study enrolment, n (%)	8 (11.9%)	22 (33.8%)	**0.27 (0.11–0.65), 0.004**	0 (-)	30 (26.5%)	0.07 (0.00–1.20), 0.067 ^
Age at study enrolment, years, mean ± SD	63.2 ± 11.9	58.0 ± 13.7	**—, 0.022**	67.3 ± 8.6	59.6 ± 13.4	**—, 0.016**
Age < 50 years at first melanoma diagnosis	19 (28.4%)	30 (46.2%)	**0.46 (0.22–0.95), 0.036**	3 (15.8%)	46 (40.7%)	**0.27 (0.08–0.99), 0.048**
Age at melanoma diagnosis, years, mean ± SD	56.5 ± 12.0	51.1 ± 15.0	**—, 0.024**	61.4 ± 9.8	52.5 ± 14.0	**—, 0.009**
Females, n (%)	20 (29.9%)	39 (60.0%)	**0.28 (0.14–0.58), <0.001**	4 (21.1%)	55 (48.7%)	**0.28 (0.09–0.90), 0.033**
Males, n (%)	47 (70.1%)	26 (40.0%)	**3.52 (1.71–7.25), <0.001**	15 (78.9%)	58 (51.3%)	**3.56 (1.11–11.38), 0.033**
Ever smoker, n (%)	38 (56.7%)	26 (40.0%)	1.97 (0.98–3.93), 0.056 ^	14 (73.7%)	50 (44.2%)	**3.53 (1.19–10.46), 0.023**
CC + ever smoker, n (%)	38 (56.7%)	10 (15.4%)	**7.21 (3.15–16.51), <0.001**	14 (73.7%)	34 (30.1%)	**6.51 (2.17–19.49), <0.001**
≥10 cigarettes/day, ever, n (%)	29 (43.3%)	20 (30.8%)	1.72 (0.84–3.51), 0.138	13 (68.4%)	36 (31.9%)	**4.63 (1.63–13.18), 0.004**
CC + ≥10 cigarettes/day, ever, n (%)	29 (43.3%)	7 (10.8%)	**6.32 (2.52–15.89), <0.001**	13 (68.4%)	23 (20.4%)	**8.48 (2.91–24.72), <0.001**
≥20 cigarettes/day, ever, n (%)	16 (23.9%)	11 (16.9%)	1.54 (0.65–3.63), 0.324	9 (47.4%)	18 (15.9%)	**4.75 (1.69–13.33), 0.003**
CC + ≥20 cigarettes/day, ever, n (%)	16 (23.9%)	3 (4.6%)	**6.48 (1.79–23.50), 0.004**	9 (47.4%)	10 (8.8%)	**9.27 (3.05–28.13), <0.001**
≥10 years smoking, ever, n (%)	34 (50.7%)	20 (30.8%)	**2.32 (1.14–4.72), 0.021**	13 (68.4%)	41 (36.3%)	**3.80 (1.34–10.77), 0.012**
CC + ≥10 years smoking, ever, n (%)	34 (50.7%)	7 (10.8%)	**8.54 (3.41–21.40), <0.001**	13 (68.4%)	28 (24.8%)	**6.58 (2.28–18.94), <0.001**
≥20 years smoking, ever, n (%)	27 (40.3%)	15 (23.1%)	**2.25 (1.06–4.79), 0.035**	11 (57.9%)	31 (27.4%)	**3.64 (1.34–9.89), 0.011**
CC + ≥20 years smoking, ever, n (%)	27 (40.3%)	5 (7.7%)	**8.10 (2.88–22.80), <0.001**	11 (57.9%)	21 (18.6%)	**6.02 (2.16–16.82), <0.001**
Pack-years, mean ± SD	12.3 ± 20.3	6.5 ± 11.2	**—, 0.044**	18.8 ± 20.8	7.9 ± 15.5	**—, 0.008**
≥10 pack-years, n (%)	28 (41.8%)	16 (24.6%)	**2.20 (1.04–4.63), 0.038**	12 (63.2%)	32 (28.3%)	**4.34 (1.57–12.01), 0.005**
CC + ≥10 pack-years, n (%)	28 (41.8%)	5 (7.7%)	**8.62 (3.07–24.22), <0.001**	12 (63.2%)	21 (18.6%)	**7.51 (2.64–21.37), <0.001**
≥20 pack-years, n (%)	17 (25.4%)	10 (15.4%)	1.87 (0.78–4.46), 0.158	7 (36.8%)	20 (17.7%)	2.71 (0.95–7.75), 0.062 ^^^
CC + ≥20 pack-years, n (%)	17 (25.4%)	2 (3.1%)	**10.71 (2.36–48.55), 0.002**	7 (36.8%)	12 (10.6%)	**4.91 (1.62–14.86), 0.005**
Alcohol drinker, n (%)	45 (67.2%)	40 (61.5%)	1.28 (0.63–2.61), 0.500	15 (78.9%)	70 (61.9%)	2.30 (0.72–7.40), 0.161
High-school diploma or university degree, n (%)	35 (52.2%)	41 (63.1%)	0.64 (0.32–1.28), 0.209	9 (47.4%)	67 (59.3%)	0.62 (0.23–1.64), 0.333
Phototype 1 and 2, n (%)	36 (53.7%)	41 (63.1%)	0.68 (0.34–1.36), 0.277	9 (47.4%)	68 (60.2%)	0.60 (0.22–1.58), 0.298
Nevi ≥ 50, n (%)	30 (44.8%)	36 (55.4%)	0.65 (0.33–1.30), 0.224	6 (31.6%)	60 (53.1%)	0.41 (0.14–1.15), 0.089 ^
Low/no tanner, n (%)	40 (59.7%)	34 (52.3%)	1.35 (0.68–2.69), 0.393	9 (47.4%)	65 (57.5%)	0.66 (0.25–1.76), 0.411
Sun burns over 5 lifelong, n (%)	38 (56.7%)	34 (52.3%)	1.19 (0.60–2.37), 0.611	8 (42.1%)	64 (56.6%)	0.56 (0.21–1.49), 0.243
Indoor tanning ≥ 1 ever, n (%)	12 (17.9%)	18 (27.7%)	0.57 (0.25–1.30), 0.183	0 (—)	30 (26.5%)	0.07 (0.00–1.20), 0.067 ^
MeM, n (%)	37 (55.2%)	28 (43.1%)	1.63 (0.82–3.24), 0.164	12 (63.2%)	53 (46.9%)	1.94 (0.71–5.29), 0.195
Stage I, n (%)	21 (31.3%)	27 (41.5%)	0.64 (0.31–1.31), 0.225	5 (26.3%)	43 (38.1%)	0.58 (0.20–1.73), 0.329
Stage II, n (%)	9 (13.4%)	10 (15.4%)	0.85 (0.32–2.26), 0.750	2 (10.5%)	17 (15.0%)	0.66 (0.14–3.14), 0.606
Stage III, n (%)	21 (31.3%)	12 (18.5%)	2.02 (0.90–4.54), 0.090 ^	10 (52.6%)	23 (20.4%)	**4.35 (1.58–11.94), 0.004**
Stage IV, n (%)	16 (23.9%)	16 (24.6%)	0.96 (0.43–2.13), 0.922	2 (10.5%)	30 (26.5%)	0.33 (0.07–1.49), 0.149
Trunk, n (%)	33 (49.3%)	42 (64.6%)	0.53 (0.26–1.07), 0.076 ^	7 (36.8%)	68 (60.2%)	0.39 (0.14–1.06), 0.064 ^
Upper limb, n (%)	5 (7.5%)	4 (6.2%)	1.23 (0.32–4.80), 0.766	2 (10.5%)	7 (6.2%)	1.78 (0.34–9.30), 0.493
Lower limb, n (%)	15 (22.4%)	12 (18.5%)	1.27 (0.54–2.98), 0.577	3 (15.8%)	24 (21.2%)	0.70 (0.19–2.58), 0.587
Hand/foot, n (%)	4 (6.0%)	4 (6.2%)	0.97 (0.23–4.05), 0.965	1 (5.3%)	7 (6.2%)	0.84 (0.10–7.25), 0.875
Head/neck, n (%)	10 (14.9%)	3 (4.6%)	3.63 (0.95–13.84), 0.059 ^	6 (31.6%)	7 (6.2%)	**6.99 (2.04–23.99), 0.002**
Ulceration, n (%)	29 (43.3%)	22 (33.8%)	1.49 (0.74–3.02), 0.267	9 (47.4%)	42 (37.2%)	1.52 (0.57–4.05), 0.400
Non-brisk ^a^ TILs, n (%)	19 (28.4%)	26 (40.0%)	0.59 (0.29–1.23), 0.160	4 (21.1%)	41 (36.3%)	0.47 (0.15–1.51), 0.203
^a^ TILs absence, n (%)	25 (37.3%)	20 (30.8%)	1.34 (0.65–2.76), 0.428	10 (52.6%)	35 (31.0%)	2.48 (0.92–6.63), 0.071 ^
Epithelioid cytological variant, n (%)	25 (37.3%)	11 (16.9%)	**2.92 (1.29–6.61), 0.010**	7 (36.8%)	29 (25.7%)	1.69 (0.61–4.70), 0.315
Fusate cytological variant, n (%)	9 (13.4%)	4 (6.2%)	2.37 (0.69–8.11), 0.170	3 (15.8%)	10 (8.8%)	1.93 (0.48–7.78), 0.355
Vascular emboli, n (%)	12 (17.9%)	3 (4.6%)	**4.51 (1.21–16.82), 0.025**	4 (21.1%)	11 (9.7%)	2.47 (0.70–8.77), 0.161
Melanoma familiarity, n (%)	11 (16.4%)	7 (10.8%)	1.63 (0.59–4.50), 0.348	3 (15.8%)	15 (13.3%)	1.23 (0.32–4.71), 0.768

Categorical variables were evaluated using ORs and CIs; continuous variables were compared using Student’s *t* test for independent samples. ^a^ TILs, tumor-infiltrating lymphocytes. Statistically significant estimates are shown in bold; trends are denoted by ^. OR and CI were not calculable for continuous variables. Not calculable values were indicated by —.

**Table 4:** Comparison of demographic and clinicopathological characteristics between melanoma patients with *HIF1A* rs11549465 CC plus ≥ 10 cigarettes/day (n = 36) and the remaining patients (n = 96), and between patients with CC plus ≥ 20 cigarettes/day (n = 19) and the remaining patients (n = 113).

Variable	CC + ≥10 Cigarettes/Day (n = 36)	Other Patients (n = 96)	OR (CI), *p*	CC + ≥20 Cigarettes/Day (n = 19)	Other Patients (n = 113)	OR (CI), *p*
Age < 50 years at study enrolment, n (%)	4 (11.1%)	26 (27.1%)	0.34 (0.11–1.04), 0.060 ^	1 (5.3%)	29 (25.7%)	0.16 (0.02–1.26), 0.082 ^
Age at study enrolment, years, mean ± SD	63.8 ± 11.4	59.5 ± 13.5	—, 0.095 ^	67.5 ± 11.6	59.5 ± 13.0	**—, 0.014**
Age < 50 years at first melanoma diagnosis	12 (33.3%)	37 (38.5%)	0.80 (0.36–1.78), 0.582	5 (26.3%)	44 (38.9%)	0.56 (0.19–1.66), 0.297
Age at melanoma diagnosis, years, mean ± SD	56.8 ± 11.9	52.7 ± 14.3	—, 0.133	60.3 ± 11.9	52.7 ± 13.8	**—, 0.025**
Females, n (%)	9 (25.0%)	50 (52.1%)	**0.31 (0.13–0.72),** **0.007**	2 (10.5%)	57 (50.4%)	**0.12 (0.03–0.52), 0.005**
Males, n (%)	27 (75.0%)	46 (47.9%)	**3.26 (1.39–7.66),** **0.007**	17 (89.5%)	56 (49.6%)	**8.65 (1.91–39.20), 0.005**
BMI ≥ 25 kg/m^2^, n (%)	29 (80.6%)	56 (58.3%)	**2.96 (1.18–7.42),** **0.021**	16 (84.2%)	69 (61.1%)	3.40 (0.94–12.35), 0.063 ^
CC + BMI ≥ 25 kg/m^2^, n (%)	29 (80.6%)	38 (39.6%)	**6.32 (2.52–15.89), <0.001**	16 (84.2%)	51 (45.1%)	**6.48 (1.79–23.50), 0.004**
BMI ≥ 30 kg/m^2^, n (%)	13 (36.1%)	10 (10.4%)	**4.86 (1.89–12.50),** **0.001**	9 (47.4%)	14 (12.4%)	**6.36 (2.20–18.37), <0.001**
CC + BMI ≥ 30 kg/m^2^, n (%)	13 (36.1%)	6 (6.2%)	**8.48 (2.91–24.72), <0.001**	9 (47.4%)	10 (8.8%)	**9.27 (3.05–28.13), <0.001**
Alcohol drinker, n (%)	30 (83.3%)	55 (57.3%)	**3.73 (1.42–9.79), 0.008**	17 (89.5%)	68 (60.2%)	**5.62 (1.24–25.53), 0.025**
High-school diploma or university degree, n (%)	20 (55.6%)	56 (58.3%)	0.89 (0.41–1.93), 0.774	9 (47.4%)	67 (59.3%)	0.62 (0.23–1.64), 0.333
Phototype 1 and 2, n (%)	22 (61.1%)	55 (57.3%)	1.17 (0.54–2.56), 0.692	12 (63.2%)	65 (57.5%)	1.27 (0.46–3.46), 0.645
Nevi ≥ 50, n (%)	20 (55.6%)	46 (47.9%)	1.36 (0.63–2.93), 0.435	9 (47.4%)	57 (50.4%)	0.88 (0.33–2.34), 0.804
Low/no tanner, n (%)	19 (52.8%)	55 (57.3%)	0.83 (0.39–1.80), 0.642	10 (52.6%)	64 (56.6%)	0.85 (0.32–2.25), 0.745
Sun burns over 5 lifelong, n (%)	21 (58.3%)	51 (53.1%)	1.24 (0.57–2.68), 0.593	13 (68.4%)	59 (52.2%)	1.98 (0.70–5.58), 0.195
Indoor tanning ≥ 1 ever, n (%)	4 (11.1%)	26 (27.1%)	0.34 (0.11–1.04), 0.060 ^	2 (10.5%)	28 (24.8%)	0.36 (0.08–1.64), 0.186
MeM, n (%)	20 (55.6%)	45 (46.9%)	1.42 (0.66–3.06), 0.375	12 (63.2%)	53 (46.9%)	1.94 (0.71–5.29), 0.195
Stage I, n (%)	14 (38.9%)	34 (35.4%)	1.16 (0.53–2.56), 0.712	5 (26.3%)	43 (38.1%)	0.58 (0.20–1.73), 0.329
Stage II, n (%)	2 (5.6%)	17 (17.7%)	0.27 (0.06–1.25), 0.094 ^	2 (10.5%)	17 (15.0%)	0.66 (0.14–3.14), 0.606
Stage III, n (%)	11 (30.6%)	22 (22.9%)	1.48 (0.63–3.48), 0.368	6 (31.6%)	27 (23.9%)	1.47 (0.51–4.24), 0.476
Stage IV, n (%)	9 (25.0%)	23 (24.0%)	1.06 (0.44–2.57), 0.901	6 (31.6%)	26 (23.0%)	1.54 (0.53–4.47), 0.422
Trunk, n (%)	14 (38.9%)	61 (63.5%)	**0.37 (0.17–0.80), 0.012**	7 (36.8%)	68 (60.2%)	0.39 (0.14–1.06), 0.064 ^
Upper limb, n (%)	2 (5.6%)	7 (7.3%)	0.75 (0.15–3.78), 0.725	1 (5.3%)	8 (7.1%)	0.73 (0.09–6.19), 0.772
Lower limb, n (%)	7 (19.4%)	20 (20.8%)	0.92 (0.35–2.40), 0.860	3 (15.8%)	24 (21.2%)	0.70 (0.19–2.58), 0.587
Hand/foot, n (%)	5 (13.9%)	3 (3.1%)	**5.00 (1.13–22.14), 0.034**	3 (15.8%)	5 (4.4%)	4.05 (0.88–18.60), 0.072 ^
Head/neck, n (%)	8 (22.2%)	5 (5.2%)	**5.20 (1.57–17.18), 0.007**	5 (26.3%)	8 (7.1%)	**4.69 (1.34–16.34), 0.015**
Ulceration, n (%)	14 (38.9%)	37 (38.5%)	1.01 (0.46–2.23), 0.971	9 (47.4%)	42 (37.2%)	1.52 (0.57–4.05), 0.400
Non-brisk ^a^ TILs, n (%)	7 (19.4%)	38 (39.6%)	**0.37 (0.15–0.93), 0.034**	1 (5.3%)	44 (38.9%)	**0.09 (0.01–0.68), 0.020**
^a^ TILs absence, n (%)	16 (44.4%)	29 (30.2%)	1.85 (0.84–4.07), 0.127	13 (68.4%)	32 (28.3%)	**5.48 (1.92–15.68), 0.001**
Epithelioid cytological variant, n (%)	14 (38.9%)	22 (22.9%)	2.14 (0.94–4.87), 0.070 ^	8 (42.1%)	28 (24.8%)	2.21 (0.81–6.04), 0.123
Fusate cytological variant, n (%)	6 (16.7%)	7 (7.3%)	2.54 (0.79–8.16), 0.117	5 (26.3%)	8 (7.1%)	**4.69 (1.34–16.34), 0.015**
Vascular emboli, n (%)	3 (8.3%)	12 (12.5%)	0.64 (0.17–2.40), 0.505	2 (10.5%)	13 (11.5%)	0.90 (0.19–4.37), 0.901
Melanoma familiarity, n (%)	8 (22.2%)	10 (10.4%)	2.46 (0.88–6.83), 0.085 ^	4 (21.1%)	14 (12.4%)	1.89 (0.55–6.50), 0.315

Categorical variables were evaluated using ORs and CIs; continuous variables were compared using Student’s *t* test for independent samples. ^a^ TILs, tumor-infiltrating lymphocytes. Statistically significant estimates are shown in bold; trends are denoted by ^. OR and CI were not calculable for continuous variables. Not calculable values were indicated by —.

**Table 5:** Comparison of demographic and clinicopathological characteristics between melanoma patients with *HIF1A* rs11549465 CC plus ≥10 years of smoking (n = 41) and the remaining patients (n = 91), and between patients with CC plus ≥20 years of smoking (n = 32) and the remaining patients (n = 100).

Variable	CC + ≥10 Years Smoking (n = 41)	Other Patients (n = 91)	OR (CI), *p*	CC + ≥20 Years Smoking (n = 32)	Other Patients (n = 100)	OR (CI), *p*
Age < 50 years at study enrolment, n (%)	5 (12.2%)	25 (27.5%)	0.37 (0.13–1.04), 0.059 ^	4 (12.5%)	26 (26.0%)	0.41 (0.13–1.27), 0.121
Age at study enrolment, years, mean ± SD	63.7 ± 12.1	59.3 ± 13.3	—, 0.073 ^	63.6 ± 11.5	59.8 ± 13.4	—, 0.152
Age < 50 years at first melanoma diagnosis	13 (31.7%)	36 (39.6%)	0.71 (0.32–1.55), 0.388	10 (31.2%)	39 (39.0%)	0.71 (0.30–1.66), 0.431
Age at melanoma diagnosis, years, mean ± SD	56.8 ± 11.8	52.5 ± 14.4	—, 0.098 ^	56.6 ± 11.3	52.9 ± 14.4	—, 0.189
Females, n (%)	10 (24.4%)	49 (53.8%)	**0.28 (0.12–0.63), 0.002**	8 (25.0%)	51 (51.0%)	**0.32 (0.13–0.78), 0.012**
Males, n (%)	31 (75.6%)	42 (46.2%)	**3.62 (1.59–8.24), 0.002**	24 (75.0%)	49 (49.0%)	**3.12 (1.28–7.61), 0.012**
BMI ≥ 25 kg/m^2^, n (%)	34 (82.9%)	51 (56.0%)	**3.81 (1.53–9.49), 0.004**	27 (84.4%)	58 (58.0%)	**3.91 (1.39–10.99), 0.010**
CC + BMI ≥ 25 kg/m^2^, n (%)	34 (82.9%)	33 (36.3%)	**8.54 (3.41–21.40), <0.001**	27 (84.4%)	40 (40.0%)	**8.10 (2.88–22.80), <0.001**
BMI ≥ 30 kg/m^2^, n (%)	13 (31.7%)	10 (11.0%)	**3.76 (1.48–9.53), 0.005**	11 (34.4%)	12 (12.0%)	**3.84 (1.49–9.90), 0.005**
CC + BMI ≥ 30 kg/m^2^, n (%)	13 (31.7%)	6 (6.6%)	**6.58 (2.28–18.94), <0.001**	11 (34.4%)	8 (8.0%)	**6.02 (2.16–16.82), <0.001**
Alcohol drinker, n (%)	33 (80.5%)	52 (57.1%)	**3.09 (1.29–7.44), 0.012**	26 (81.2%)	59 (59.0%)	**3.01 (1.14–7.97), 0.026**
High-school diploma or university degree, n (%)	25 (61.0%)	51 (56.0%)	1.23 (0.58–2.60), 0.596	20 (62.5%)	56 (56.0%)	1.31 (0.58–2.97), 0.518
Phototype 1 and 2, n (%)	25 (61.0%)	52 (57.1%)	1.17 (0.55–2.49), 0.679	17 (53.1%)	60 (60.0%)	0.76 (0.34–1.68), 0.493
Nevi ≥ 50, n (%)	21 (51.2%)	45 (49.5%)	1.07 (0.51–2.24), 0.851	18 (56.2%)	48 (48.0%)	1.39 (0.63–3.10), 0.418
Low/no tanner, n (%)	22 (53.7%)	52 (57.1%)	0.87 (0.41–1.82), 0.709	16 (50.0%)	58 (58.0%)	0.72 (0.33–1.61), 0.428
Sun burns over 5 lifelong, n (%)	26 (63.4%)	46 (50.5%)	1.70 (0.80–3.61), 0.171	19 (59.4%)	53 (53.0%)	1.30 (0.58–2.91), 0.529
Indoor tanning ≥ 1 ever, n (%)	6 (14.6%)	24 (26.4%)	0.48 (0.18–1.28), 0.142	6 (18.8%)	24 (24.0%)	0.73 (0.27–1.99), 0.538
MeM, n (%)	25 (61.0%)	40 (44.0%)	1.99 (0.94–4.22), 0.072 ^	21 (65.6%)	44 (44.0%)	**2.43 (1.06–5.57), 0.036**
Stage I, n (%)	13 (31.7%)	35 (38.5%)	0.74 (0.34–1.62), 0.456	9 (28.1%)	39 (39.0%)	0.61 (0.26–1.46), 0.268
Stage II, n (%)	3 (7.3%)	16 (17.6%)	0.37 (0.10–1.35), 0.132	2 (6.2%)	17 (17.0%)	0.33 (0.07–1.49), 0.149
Stage III, n (%)	13 (31.7%)	20 (22.0%)	1.65 (0.72–3.76), 0.235	13 (40.6%)	20 (20.0%)	**2.74 (1.16–6.46), 0.022**
Stage IV, n (%)	12 (29.3%)	20 (22.0%)	1.47 (0.64–3.39), 0.367	8 (25.0%)	24 (24.0%)	1.06 (0.42–2.66), 0.909
Trunk, n (%)	18 (43.9%)	57 (62.6%)	**0.47 (0.22–0.99), 0.046**	14 (43.8%)	61 (61.0%)	0.50 (0.22–1.11), 0.089 ^
Upper limb, n (%)	2 (4.9%)	7 (7.7%)	0.62 (0.12–3.10), 0.556	2 (6.2%)	7 (7.0%)	0.89 (0.17–4.50), 0.884
Lower limb, n (%)	8 (19.5%)	19 (20.9%)	0.92 (0.36–2.31), 0.857	6 (18.8%)	21 (21.0%)	0.87 (0.32–2.38), 0.784
Hand/foot, n (%)	5 (12.2%)	3 (3.3%)	4.07 (0.92–17.95), 0.063 ^	3 (9.4%)	5 (5.0%)	1.97 (0.44–8.73), 0.374
Head/neck, n (%)	8 (19.5%)	5 (5.5%)	**4.17 (1.27–13.67), 0.018**	7 (21.9%)	6 (6.0%)	**4.39 (1.35–14.22), 0.014**
Ulceration, n (%)	18 (43.9%)	33 (36.3%)	1.38 (0.65–2.91), 0.405	15 (46.9%)	36 (36.0%)	1.57 (0.70–3.51), 0.273
Non-brisk ^a^ TILs, n (%)	8 (19.5%)	37 (40.7%)	**0.35 (0.15–0.85), 0.020**	6 (18.8%)	39 (39.0%)	**0.36 (0.14–0.96), 0.040**
^a^ TILs absence, n (%)	19 (46.3%)	26 (28.6%)	**2.16 (1.01–4.64), 0.048**	14 (43.8%)	31 (31.0%)	1.73 (0.76–3.92), 0.188
Epithelioid cytological variant, n (%)	16 (39.0%)	20 (22.0%)	**2.27 (1.02–5.06), 0.044**	13 (40.6%)	23 (23.0%)	2.29 (0.98–5.33), 0.055 ^
Fusate cytological variant, n (%)	6 (14.6%)	7 (7.7%)	2.06 (0.65–6.56), 0.223	6 (18.8%)	7 (7.0%)	3.07 (0.95–9.92), 0.061 ^
Vascular emboli, n (%)	4 (9.8%)	11 (12.1%)	0.79 (0.23–2.63), 0.697	3 (9.4%)	12 (12.0%)	0.76 (0.20–2.88), 0.685
Melanoma familiarity, n (%)	8 (19.5%)	10 (11.0%)	1.96 (0.71–5.41), 0.192	4 (12.5%)	14 (14.0%)	0.88 (0.27–2.89), 0.830

Categorical variables were evaluated using ORs and CIs; continuous variables were compared using Student’s *t* test for independent samples. ^a^ TILs, tumor-infiltrating lymphocytes. Statistically significant estimates are shown in bold; trends are denoted by ^. OR and CI were not calculable for continuous variables. Not calculable values were indicated by —.

**Table 6:** Comparison of *HIF1A* rs11549465 (1772 C>T), demographic, lifestyle, and clinicopathological characteristics between patients with head/neck melanoma (n = 13) and patients with melanoma at other sites (n = 119), with age- and sex-adjusted comparisons against 312 healthy controls.

Variable	Head/Neck Melanoma (n = 13)	Other Sites (n = 119)	OR (CI), *p* Head/Neck vs. Other Sites	aOR (CI), *p* Head/Neck vs. Healthy Controls
Age < 50 years at study enrolment, n (%)	0 (—)	30 (25.2%)	0.11 (0.01–1.88), 0.127	—
Age at study enrolment, years, mean ± SD	68.8 ± 9.7	59.8 ± 13.1	**—, 0.017**	—
Age < 50 years at first melanoma diagnosis, n (%)	0 (—)	49 (41.2%)	**0.05 (0.00–0.91), 0.043**	—
Age at melanoma diagnosis, years, mean ± SD	64.3 ± 10.1	52.7 ± 13.7	**—, 0.003**	—
Females, n (%)	3 (23.1%)	56 (47.1%)	0.34 (0.09–1.29), 0.112	—
Males, n (%)	10 (76.9%)	63 (52.9%)	2.96 (0.78–11.31), 0.112	—
BMI ≥ 25 kg/m^2^, n (%)	10 (76.9%)	75 (63.0%)	1.96 (0.51–7.49), 0.328	2.55 (0.63–10.30), 0.188
CC + BMI ≥ 25 kg/m^2^, n (%)	10 (76.9%)	57 (47.9%)	3.63 (0.95–13.84), 0.059 ^	**5.00 (1.27–19.69), 0.022**
BMI ≥ 30 kg/m^2^, n (%)	6 (46.2%)	17 (14.3%)	**5.14 (1.54–17.17), 0.008**	**5.55 (1.67–18.40), 0.005**
CC + BMI ≥ 30 kg/m^2^, n (%)	6 (46.2%)	13 (10.9%)	**6.99 (2.04–23.99), 0.002**	**9.07 (2.60–31.59), <0.001**
Ever smoker, n (%)	8 (61.5%)	56 (47.1%)	1.80 (0.56–5.82), 0.326	1.52 (0.45–5.07), 0.499
CC + ever smoker, n (%)	8 (61.5%)	40 (33.6%)	3.16 (0.97–10.29), 0.056 ^	2.66 (0.81–8.72), 0.107
Past smoker, n (%)	8 (61.5%)	43 (36.1%)	2.83 (0.87–9.19), 0.084 ^	**4.43 (1.27–15.40), 0.019**
CC + past smoker, n (%)	8 (61.5%)	32 (26.9%)	**4.35 (1.33–14.28), 0.015**	**6.11 (1.78–20.97), 0.004**
≥10 cigarettes/day, ever, n (%)	8 (61.5%)	41 (34.5%)	3.04 (0.94–9.90), 0.064 ^	**3.96 (1.22–12.86), 0.022**
CC + ≥10 cigarettes/day, ever, n (%)	8 (61.5%)	28 (23.5%)	**5.20 (1.57–17.18), 0.007**	**5.50 (1.71–17.75), 0.004**
≥20 cigarettes/day, ever, n (%)	5 (38.5%)	22 (18.5%)	2.76 (0.82–9.24), 0.100	**4.44 (1.31–15.02), 0.017**
CC + ≥20 cigarettes/day, ever, n (%)	5 (38.5%)	14 (11.8%)	**4.69 (1.34–16.34), 0.015**	**5.96 (1.74–20.38), 0.004**
≥10 years smoking, ever, n (%)	8 (61.5%)	46 (38.7%)	2.54 (0.78–8.24), 0.121	2.25 (0.69–7.39), 0.181
CC + ≥10 years smoking, ever, n (%)	8 (61.5%)	33 (27.7%)	**4.17 (1.27–13.67), 0.018**	**3.41 (1.05–11.05), 0.041**
≥20 years smoking, ever, n (%)	7 (53.8%)	35 (29.4%)	2.80 (0.88–8.93), 0.082 ^	**4.39 (1.31–14.67), 0.016**
CC + ≥20 years smoking, ever, n (%)	7 (53.8%)	25 (21.0%)	**4.39 (1.35–14.22), 0.014**	**6.64 (1.94–22.71), 0.003**
Pack-years, mean ± SD	14.9 ± 17.7	8.8 ± 16.5	—, 0.214	—
≥10 pack-years, n (%)	8 (61.5%)	36 (30.3%)	**3.69 (1.13–12.05), 0.031**	**5.37 (1.61–17.87), 0.006**
CC + ≥10 pack-years, n (%)	8 (61.5%)	25 (21.0%)	**6.02 (1.81–20.00), 0.003**	**7.21 (2.18–23.87), 0.001**
≥20 pack-years, n (%)	4 (30.8%)	23 (19.3%)	1.86 (0.52–6.56), 0.337	3.82 (0.97–14.96), 0.055 ^
CC + ≥20 pack-years, n (%)	4 (30.8%)	15 (12.6%)	3.08 (0.84–11.26), 0.089 ^	**5.01 (1.24–20.19), 0.024**
Alcohol drinker, n (%)	9 (69.2%)	76 (63.9%)	1.27 (0.37–4.38), 0.702	—
High-school diploma or university degree, n (%)	7 (53.8%)	69 (58.0%)	0.85 (0.27–2.67), 0.775	—
Phototype 1 and 2, n (%)	5 (38.5%)	72 (60.5%)	0.41 (0.13–1.32), 0.135	—
Nevi ≥ 50, n (%)	3 (23.1%)	63 (52.9%)	0.27 (0.07–1.02), 0.053 ^	—
Low/no tanner, n (%)	5 (38.5%)	69 (58.0%)	0.45 (0.14–1.47), 0.186	—
Sun burns over 5 lifelong, n (%)	4 (30.8%)	68 (57.1%)	0.33 (0.10–1.14), 0.081 ^	—
Indoor tanning ≥ 1 ever, n (%)	3 (23.1%)	27 (22.7%)	1.02 (0.26–3.98), 0.975	—
MeM, n (%)	9 (69.2%)	56 (47.1%)	2.53 (0.74–8.67), 0.139	—
Stage I, n (%)	3 (23.1%)	45 (37.8%)	0.49 (0.13–1.89), 0.302	—
Stage II, n (%)	1 (7.7%)	18 (15.1%)	0.47 (0.06–3.82), 0.478	—
Stage III, n (%)	3 (23.1%)	30 (25.2%)	0.89 (0.23–3.45), 0.866	—
Stage IV, n (%)	6 (46.2%)	26 (21.8%)	3.07 (0.95–9.92), 0.061 ^	—
Ulceration, n (%)	9 (69.2%)	42 (35.3%)	**4.12 (1.20–14.2), 0.025**	—
Non-brisk ^a^ TILs, n (%)	2 (15.4%)	43 (36.1%)	0.32 (0.07–1.52), 0.152	—
^a^ TILs absence, n (%)	6 (46.2%)	39 (32.8%)	1.76 (0.55–5.58), 0.339	—
Epithelioid cytological variant, n (%)	3 (23.1%)	33 (27.7%)	0.78 (0.20–3.02), 0.721	—
Fusate cytological variant, n (%)	2 (15.4%)	11 (9.2%)	1.79 (0.35–9.11), 0.486	—
Vascular emboli, n (%)	1 (7.7%)	14 (11.8%)	0.62 (0.08–5.18), 0.663	—
Melanoma familiarity, n (%)	1 (7.7%)	17 (14.3%)	0.50 (0.06–4.10), 0.518	—

Categorical variables were evaluated using ORs and CIs; continuous variables were compared using Student’s *t* test for independent samples. Adjusted ORs include age and sex. ^a^ TILs, tumor-infiltrating lymphocytes. Statistically significant estimates are shown in bold; trends are denoted by ^. OR and CI were not calculable for continuous variables. Not calculable values were indicated by —.

## Data Availability

All data from this study are available upon reasonable request.

## Supplementary Materials

The following supporting information can be downloaded at: https://www.mdpi.com/article/10.3390/cancers18172787/s1, Table S1: Genotype/allele frequencies, BMI-variables, and selected histological and clinicopathological characteristics corresponding to Table 3: melanoma patients with *HIF1A* rs11549465 CC plus BMI ≥ 25 kg/m^2^ (n = 67) versus other patients (n = 65), and CC plus BMI ≥ 30 kg/m^2^ (n = 19) versus other patients (n = 113); Table S2: Genotype/allele frequencies, smoking-variables, and selected histological and clinicopathological characteristics corresponding to Table 4: melanoma patients with *HIF1A* rs11549465 CC plus ≥ 10 cigarettes/day (n = 36) versus other patients (n = 96), and CC plus ≥ 20 cigarettes/day (n = 19) versus other patients (n = 113); Table S3: Genotype/allele frequencies, smoking-variables, and selected histological and clinicopathological characteristics corresponding to Table 5: melanoma patients with *HIF1A* rs11549465 CC plus ≥ 10 years of smoking (n = 41) versus other patients (n = 91), and CC plus ≥ 20 years of smoking (n = 32) versus other patients (n = 100); Table S4: Genotype/allele frequencies, smoking-variables, and selected histological and clinicopathological characteristics corresponding to Table 6: patients with head/neck melanoma (n = 13) versus patients with melanoma at other sites (n = 119).
