# Supplementary material for: Hypoxia-Inducible Factor-1α Gene (HIF1A) rs11549465 (1772 C>T) Polymorphism, Body Mass Index, Smoking, and Cutaneous Melanoma: An Observational Case–Control Study
Source: Cancers (Basel). 2026 Aug 27;18(17):2787. doi: 10.3390/cancers18172787 (PMC13565261; doi:10.3390/cancers18172787)
Supplement: Supplementary file 1 [file cancers-18-02787-s001.zip › cancers-4500320-supplementary.pdf]

Supplementary Tables

Supplementary Table S1. Genotype/allele frequencies, BMI-variables, and selected histological and clinicopathological characteristics corresponding to Table 3: melanoma patients with *HIF1A* rs11549465 CC plus BMI ≥25 kg/m² (n=67) versus other patients (n=65), and CC plus BMI ≥30 kg/m² (n=19) versus other patients (n=113).

Variable	CC + BMI ⩾25 kg/m² (n=67)	Other patients (n=65)	OR (CI), P	CC + BMI ⩾30 kg/m² (n=19)	Other patients (n=113)	OR (CI), P
TT genotype, n (%)	0 (-)	2 (3.1%)	0.19 (0.01-4.00), 0.284	0 (-)	2 (1.8%)	1.14 (0.05-24.74), 0.932
CT genotype, n (%)	0 (-)	31 (47.7%)	**0.01 (0.00-0.14), <0.001**	0 (-)	31 (27.4%)	0.07 (0.00-1.15), 0.062^
CC genotype, n (%)	67 (100.0%)	32 (49.2%)	**139.1 (8.26-2343), <0.001**	19 (100.0%)	80 (70.8%)	16.23 (0.95-276.66), 0.054^
T allele, n (%)	0/134 (-)	35/130 (26.9%)	**0.01 (0.00-0.17), 0.001**	0/38 (-)	35/226 (15.5%)	0.07 (0.00-1.17), 0.064^
C allele, n (%)	134/134 (100.0%)	95/130 (73.1%)	**99.99 (6.06-1650), 0.001**	38/38 (100.0%)	191/226 (84.5%)	14.27 (0.86-237.73), 0.064^
BMI ⩾25 kg/m², n (%)	67 (100.0%)	18 (27.7%)	**346.6 (20.38-5894), <0.001**	19 (100.0%)	66 (58.4%)	**27.86 (1.64-472.9), 0.021**
CC + BMI ⩾25 kg/m², n (%)	-	-	-	19 (100.0%)	48 (42.5%)	**52.67 (3.10-894.0), 0.006**
BMI ⩾30 kg/m², n (%)	19 (28.4%)	4 (6.2%)	**6.04 (1.93-18.92), 0.002**	19 (100.0%)	4 (3.5%)	**949.0 (49.11-18337), <0.001**
CC + BMI ⩾30 kg/m², n (%)	19 (28.4%)	0 (-)	**52.67 (3.10-894.0), 0.006**	-	-	-
Superficial spreading, n (%)	34 (50.7%)	33 (50.8%)	1.00 (0.50-1.98), 0.998	13 (68.4%)	54 (47.8%)	2.37 (0.84-6.67), 0.103
Nodular, n (%)	26 (38.8%)	21 (32.3%)	1.33 (0.65-2.72), 0.436	3 (15.8%)	44 (38.9%)	0.29 (0.08-1.07), 0.063^
Acral lentiginous, n (%)	2 (3.0%)	3 (4.6%)	0.64 (0.10-3.94), 0.626	0 (-)	5 (4.4%)	0.51 (0.03-9.52), 0.649
Lentigo maligna, n (%)	0 (-)	2 (3.1%)	0.19 (0.01-4.00), 0.284	0 (-)	2 (1.8%)	1.14 (0.05-24.74), 0.932
Spitzoide, n (%)	1 (1.5%)	4 (6.2%)	0.23 (0.03-2.12), 0.196	0 (-)	5 (4.4%)	0.51 (0.03-9.52), 0.649
Nevoid malignant, n (%)	2 (3.0%)	0 (-)	5.00 (0.24-106.2), 0.302	1 (5.3%)	1 (0.9%)	6.22 (0.37-104.0), 0.203
Breslow thickness, mm, mean±SD	2.4±1.9	1.8±1.8	-, 0.076^	2.6±1.7	2.0±1.9	-, 0.193
Breslow thickness 1.01-2.00 mm, n (%)	15 (22.4%)	23 (35.4%)	0.53 (0.24-1.13), 0.101	3 (15.8%)	35 (31.0%)	0.42 (0.11-1.53), 0.187
Breslow thickness 2.01-4.00 mm, n (%)	19 (28.4%)	11 (16.9%)	1.94 (0.84-4.49), 0.120	6 (31.6%)	24 (21.2%)	1.71 (0.59-4.98), 0.324
Breslow thickness ⩾4.01 mm, n (%)	12 (17.9%)	6 (9.2%)	2.15 (0.75-6.11), 0.153	5 (26.3%)	13 (11.5%)	2.75 (0.85-8.88), 0.091^
Mitosis ⩾1 per mm², n (%)	43 (64.2%)	42 (64.6%)	0.98 (0.48-2.00), 0.958	12 (63.2%)	73 (64.6%)	0.94 (0.34-2.58), 0.903
Regression, n (%)	6 (9.0%)	13 (20.0%)	0.39 (0.14-1.11), 0.078^	3 (15.8%)	16 (14.2%)	1.14 (0.30-4.35), 0.852
Brisk positive TILs[a], n (%)	22 (32.8%)	17 (26.2%)	1.38 (0.65-2.93), 0.401	5 (26.3%)	34 (30.1%)	0.83 (0.28-2.49), 0.739
Microsatellitosis, n (%)	4 (6.0%)	1 (1.5%)	4.06 (0.44-37.37), 0.216	2 (10.5%)	3 (2.7%)	4.31 (0.67-27.73), 0.124

Categorical variables were evaluated using ORs and CIs; continuous variables were compared using Student's t test for independent samples. [a]TILs, tumor-infiltrating lymphocytes. Statistically significant estimates are shown in bold; trends are denoted by ^.

Supplementary Table S2. Genotype/allele frequencies, smoking-variables, and selected histological and clinicopathological characteristics corresponding to Table 4: melanoma patients with *HIF1A* rs11549465 CC plus ≥10 cigarettes/day (n=36) versus other patients (n=96), and CC plus ≥20 cigarettes/day (n=19) versus other patients (n=113).

Variable	CC + ⩾10 cigarettes/day (n=36)	Other patients (n=96)	OR (CI), P	CC + ⩾20 cigarettes/day (n=19)	Other patients (n=113)	OR (CI), P
TT genotype, n (%)	0 (-)	2 (2.1%)	0.52 (0.02-11.05), 0.673	0 (-)	2 (1.8%)	1.14 (0.05-24.74), 0.932
CT genotype, n (%)	0 (-)	31 (32.3%)	**0.03 (0.00-0.48), 0.013**	0 (-)	31 (27.4%)	0.07 (0.00-1.15), 0.062^
CC genotype, n (%)	36 (100.0%)	63 (65.6%)	**38.51 (2.29-647.4), 0.011**	19 (100.0%)	80 (70.8%)	16.23 (0.95-276.7), 0.054^
T allele, n (%)	0/72 (-)	35/192 (18.2%)	**0.03 (0.00-0.51), 0.015**	0/38 (-)	35/226 (15.5%)	0.07 (0.00-1.17), 0.064^
C allele, n (%)	72/72 (100.0%)	157/192 (81.8%)	**32.68 (1.98-540.2), 0.015**	38/38 (100.0%)	191/226 (84.5%)	14.27 (0.86-237.73), 0.064^
Ever smoker, n (%)	36 (100.0%)	28 (29.2%)	**175.5 (10.41-2958), <0.001**	19 (100.0%)	45 (39.8%)	**58.71 (3.46-997.0), 0.005**
CC + ever smoker, n (%)	36 (100.0%)	12 (12.5%)	**493.5 (28.45-8559), <0.001**	19 (100.0%)	29 (25.7%)	**111.71 (6.54-1909), 0.001**
⩾10 cigarettes/day, ever, n (%)	36 (100.0%)	13 (13.5%)	**451.5 (26.13-7801), <0.001**	19 (100.0%)	30 (26.5%)	**106.8 (6.25-1823), 0.001**
CC + ⩾10 cigarettes/day, ever, n (%)	-	-	-	19 (100.0%)	17 (15.0%)	**215.1 (12.40-3729), <0.001**
⩾20 cigarettes/day, ever, n (%)	19 (52.8%)	8 (8.3%)	**12.29 (4.63-32.62), <0.001**	19 (100.0%)	8 (7.1%)	**484.1 (26.83-8735), <0.001**
CC + ⩾20 cigarettes/day, ever, n (%)	19 (52.8%)	0 (-)	**215.1 (12.40-3729), <0.001**	-	-	-
⩾10 years smoking, ever, n (%)	34 (94.4%)	20 (20.8%)	**64.60 (14.29-292.1), <0.001**	19 (100.0%)	35 (31.0%)	**86.24 (5.06-1469), 0.002**
CC + ⩾10 years smoking, ever, n (%)	34 (94.4%)	7 (7.3%)	**216.1 (42.76-1093), <0.001**	19 (100.0%)	22 (19.5%)	**158.6 (9.22-2728), <0.001**
⩾20 years smoking, ever, n (%)	27 (75.0%)	15 (15.6%)	**16.20 (6.37-41.23), <0.001**	15 (78.9%)	27 (23.9%)	**11.94 (3.65-39.05), <0.001**
CC + ⩾20 years smoking, ever, n (%)	27 (75.0%)	5 (5.2%)	**54.60 (16.87-176.7), <0.001**	15 (78.9%)	17 (15.0%)	**21.18 (6.27-71.56), <0.001**
Pack-years, mean±SD	24.9±22.0	3.6±9.0	**-, <0.001**	34.4±26.2	5.2±9.6	**-, <0.001**
⩾10 pack-years, n (%)	33 (91.7%)	11 (11.5%)	**85.00 (22.29-324.1), <0.001**	19 (100.0%)	25 (22.1%)	**135.4 (7.90-2320), <0.001**
CC + ⩾10 pack-years, n (%)	33 (91.7%)	0 (-)	**1847 (92.97-36705), <0.001**	19 (100.0%)	14 (12.4%)	**267.6 (15.32-4676), <0.001**
⩾20 pack-years, n (%)	19 (52.8%)	8 (8.3%)	**12.29 (4.63-32.62), <0.001**	15 (78.9%)	12 (10.6%)	**31.56 (9.00-110.7), <0.001**
CC + ⩾20 pack-years, n (%)	19 (52.8%)	0 (-)	**215.1 (12.40-3729), <0.001**	15 (78.9%)	4 (3.5%)	**102.2 (23.09-452.2), <0.001**
Superficial spreading, n (%)	18 (50.0%)	49 (51.0%)	0.96 (0.45-2.06), 0.915	9 (47.4%)	58 (51.3%)	0.85 (0.32-2.26), 0.750
Nodular, n (%)	12 (33.3%)	35 (36.5%)	0.87 (0.39-1.96), 0.739	6 (31.6%)	41 (36.3%)	0.81 (0.29-2.29), 0.692
Acral lentiginous, n (%)	2 (5.6%)	3 (3.1%)	1.82 (0.29-11.39), 0.520	1 (5.3%)	4 (3.5%)	1.51 (0.16-14.33), 0.718
Lentigo maligna, n (%)	0 (-)	2 (2.1%)	0.52 (0.02-11.05), 0.673	0 (-)	2 (1.8%)	1.14 (0.05-24.74), 0.932
Spitzoide, n (%)	0 (-)	5 (5.2%)	0.23 (0.01-4.23), 0.321	0 (-)	5 (4.4%)	0.51 (0.03-9.52), 0.649

Variable	CC + ⩾10 cigarettes/day (n=36)	Other patients (n=96)	OR (CI), P	CC + ⩾20 cigarettes/day (n=19)	Other patients (n=113)	OR (CI), P
Nevoid malignant, n (%)	2 (5.6%)	0 (-)	13.99 (0.65-298.6), 0.091^	1 (5.3%)	1 (0.9%)	6.22 (0.37-104.0), 0.203
Breslow thickness, mm, mean±SD	2.0±1.7	2.1±1.9	-, 0.868	2.3±1.4	2.0±1.9	-, 0.562
Breslow thickness 1.01-2.00 mm, n (%)	10 (27.8%)	28 (29.2%)	0.93 (0.40-2.19), 0.875	5 (26.3%)	33 (29.2%)	0.87 (0.29-2.60), 0.797
Breslow thickness 2.01-4.00 mm, n (%)	8 (22.2%)	22 (22.9%)	0.96 (0.38-2.41), 0.932	7 (36.8%)	23 (20.4%)	2.28 (0.81-6.45), 0.119
Breslow thickness ⩾4.01 mm, n (%)	5 (13.9%)	13 (13.5%)	1.03 (0.34-3.13), 0.959	3 (15.8%)	15 (13.3%)	1.23 (0.32-4.71), 0.768
Mitosis ⩾1 per mm^2, n (%)	23 (63.9%)	62 (64.6%)	0.97 (0.44-2.16), 0.941	11 (57.9%)	74 (65.5%)	0.72 (0.27-1.95), 0.524
Regression, n (%)	3 (8.3%)	16 (16.7%)	0.45 (0.12-1.66), 0.234	2 (10.5%)	17 (15.0%)	0.66 (0.14-3.14), 0.606
Brisk positive TILs[a], n (%)	12 (33.3%)	27 (28.1%)	1.28 (0.56-2.91), 0.560	4 (21.1%)	35 (31.0%)	0.59 (0.18-1.92), 0.384
Microsatellitosis, n (%)	2 (5.6%)	3 (3.1%)	1.82 (0.29-11.39), 0.520	2 (10.5%)	3 (2.7%)	4.31 (0.67-27.73), 0.124

Categorical variables were evaluated using ORs and CIs; continuous variables were compared using Student's t test for independent samples. [a]TILs, tumor-infiltrating lymphocytes. Statistically significant estimates are shown in bold; trends are denoted by ^.

Supplementary Table S3. Genotype/allele frequencies, smoking-variables, and selected histological and clinicopathological characteristics corresponding to Table 5: melanoma patients with *HIF1A* rs11549465 CC plus ≥10 years of smoking (n=41) versus other patients (n=91), and CC plus ≥20 years of smoking (n=32) versus other patients (n=100).

**Variable**	**CC + ⩾10 years smoking (n=41)**	**Other patients (n=91)**	**OR (CI), P**	**CC + ⩾20 years smoking (n=32)**	**Other patients (n=100)**	**OR (CI), P**
TT genotype, n (%)	0 (-)	2 (2.2%)	0.43 (0.02-9.19), 0.590	0 (-)	2 (2.0%)	0.61 (0.03-12.96), 0.749
CT genotype, n (%)	0 (-)	31 (34.1%)	**0.02 (0.00-0.39), 0.009**	0 (-)	31 (31.0%)	**0.03 (0.00-0.57), 0.019**
CC genotype, n (%)	41 (100.0%)	58 (63.7%)	**47.53 (2.83-798), 0.007**	32 (100.0%)	67 (67.0%)	**32.26 (1.92-543), 0.016**
T allele, n (%)	0/82 (-)	35/182 (19.2%)	**0.03 (0.00-0.42), 0.010**	0/64 (-)	35/200 (17.5%)	**0.04 (0.00-0.60), 0.020**
C allele, n (%)	82/82 (100.0%)	147/182 (80.8%)	**39.71 (2.40-656), 0.010**	64/64 (100.0%)	165/200 (82.5%)	**27.67 (1.67-458), 0.020**
Ever smoker, n (%)	41 (100.0%)	23 (25.3%)	**241.9 (14.31-4090), <0.001**	32 (100.0%)	32 (32.0%)	**137.0 (8.13-2308), <0.001**
CC + ever smoker, n (%)	41 (100.0%)	7 (7.7%)	**935.1 (52.14-16772), <0.001**	32 (100.0%)	16 (16.0%)	**332.9 (19.40-5712), <0.001**
⩾10 cigarettes/day, ever, n (%)	34 (82.9%)	15 (16.5%)	**24.61 (9.20-65.84), <0.001**	27 (84.4%)	22 (22.0%)	**19.15 (6.60-55.54), <0.001**
CC + ⩾10 cigarettes/day, ever, n (%)	34 (82.9%)	2 (2.2%)	**216.1 (42.76-1093), <0.001**	27 (84.4%)	9 (9.0%)	**54.60 (16.87-176.7), <0.001**
⩾20 cigarettes/day, ever, n (%)	19 (46.3%)	8 (8.8%)	**8.96 (3.46-23.18), <0.001**	15 (46.9%)	12 (12.0%)	**6.47 (2.58-16.23), <0.001**
CC + ⩾20 cigarettes/day, ever, n (%)	19 (46.3%)	0 (-)	**158.6 (9.22-2728), <0.001**	15 (46.9%)	4 (4.0%)	**21.18 (6.27-71.56), <0.001**
⩾10 years smoking, ever, n (%)	41 (100.0%)	13 (14.3%)	**482.6 (27.98-8324), <0.001**	32 (100.0%)	22 (22.0%)	**226.8 (13.35-3851), <0.001**
CC + ⩾10 years smoking, ever, n (%)	-	-	-	32 (100.0%)	9 (9.0%)	**626.0 (35.43-11063), <0.001**
⩾20 years smoking, ever, n (%)	32 (78.0%)	10 (11.0%)	**28.80 (10.71-77.44), <0.001**	32 (100.0%)	10 (10.0%)	**560.2 (31.92-9834), <0.001**
CC + ⩾20 years smoking, ever, n (%)	32 (78.0%)	0 (-)	**626.0 (35.43-11063), <0.001**	-	-	-
Pack-years, mean±SD	22.4±21.7	3.6±9.3	**-, <0.001**	26.0±23.2	4.2±9.1	**-, <0.001**
⩾10 pack-years, n (%)	33 (80.5%)	11 (12.1%)	**30.00 (11.07-81.30), <0.001**	27 (84.4%)	17 (17.0%)	**26.36 (8.89-78.23), <0.001**
CC + ⩾10 pack-years, n (%)	33 (80.5%)	0 (-)	**721.2 (40.50-12843), <0.001**	27 (84.4%)	6 (6.0%)	**84.60 (23.96-298.7), <0.001**
⩾20 pack-years, n (%)	19 (46.3%)	8 (8.8%)	**8.96 (3.46-23.18), <0.001**	19 (59.4%)	8 (8.0%)	**16.81 (6.12-46.14), <0.001**
CC + ⩾20 pack-years, n (%)	19 (46.3%)	0 (-)	**158.6 (9.22-2728), <0.001**	19 (59.4%)	0 (-)	**290.3 (16.56-5090), <0.001**
Superficial spreading, n (%)	19 (46.3%)	48 (52.7%)	0.77 (0.37-1.62), 0.496	14 (43.8%)	53 (53.0%)	0.69 (0.31-1.54), 0.364
Nodular, n (%)	16 (39.0%)	31 (34.1%)	1.24 (0.58-2.66), 0.582	13 (40.6%)	34 (34.0%)	1.33 (0.59-3.01), 0.496
Acral lentiginous, n (%)	2 (4.9%)	3 (3.3%)	1.50 (0.24-9.36), 0.662	1 (3.1%)	4 (4.0%)	0.77 (0.08-7.19), 0.822
Lentigo maligna, n (%)	0 (-)	2 (2.2%)	0.43 (0.02-9.19), 0.590	0 (-)	2 (2.0%)	0.61 (0.03-12.96), 0.749
Spitzoide, n (%)	0 (-)	5 (5.5%)	0.19 (0.01-3.51), 0.264	0 (-)	5 (5.0%)	0.27 (0.01-4.96), 0.376
Nevoid malignant, n (%)	2 (4.9%)	0 (-)	11.58 (0.54-246.8), 0.117	2 (6.2%)	0 (-)	16.48 (0.77-352.5), 0.073^
Breslow thickness, mm, mean±SD	2.2±1.7	2.0±1.9	-, 0.543	2.4±1.8	2.0±1.9	-, 0.279
Breslow thickness 1.01-2.00 mm, n (%)	11 (26.8%)	27 (29.7%)	0.87 (0.38-1.98), 0.739	6 (18.8%)	32 (32.0%)	0.49 (0.18-1.31), 0.155

Variable	CC + ⩾10 years smoking (n=41)	Other patients (n=91)	OR (CI), P	CC + ⩾20 years smoking (n=32)	Other patients (n=100)	OR (CI), P
Breslow thickness 2.01-4.00 mm, n (%)	9 (22.0%)	21 (23.1%)	0.94 (0.39-2.27), 0.886	8 (25.0%)	22 (22.0%)	1.18 (0.47-2.99), 0.725
Breslow thickness ⩾4.01 mm, n (%)	8 (19.5%)	10 (11.0%)	1.96 (0.71-5.41), 0.192	7 (21.9%)	11 (11.0%)	2.27 (0.80-6.45), 0.126
Mitosis ⩾1 per mm^2, n (%)	26 (63.4%)	59 (64.8%)	0.94 (0.44-2.03), 0.875	20 (62.5%)	65 (65.0%)	0.90 (0.39-2.05), 0.797
Regression, n (%)	3 (7.3%)	16 (17.6%)	0.37 (0.10-1.35), 0.132	2 (6.2%)	17 (17.0%)	0.33 (0.07-1.49), 0.149
Brisk positive TILs[a], n (%)	12 (29.3%)	27 (29.7%)	0.98 (0.44-2.20), 0.963	11 (34.4%)	28 (28.0%)	1.35 (0.58-3.15), 0.492
Microsatellitosis, n (%)	3 (7.3%)	2 (2.2%)	3.51 (0.56-21.88), 0.178	2 (6.2%)	3 (3.0%)	2.16 (0.34-13.51), 0.412

Categorical variables were evaluated using ORs and CIs; continuous variables were compared using Student's t test for independent samples. [a]TILs, tumor-infiltrating lymphocytes. Statistically significant estimates are shown in bold; trends are denoted by ^.

Supplementary Table S4. Genotype/allele frequencies, smoking-variables, and selected histological and clinicopathological characteristics corresponding to Table 6: patients with head/neck melanoma (n=13) versus patients with melanoma at other sites (n=119).

Variable	Head/neck melanoma (n=13)	Other sites (n=119)	OR (CI), P Head/neck vs. other sites	aOR (CI), P Head/neck vs. healthy controls
TT genotype, n (%)	0 (-)	2 (1.7%)	1.74 (0.08-38.19), 0.725	NE
CT genotype, n (%)	0 (-)	31 (26.1%)	0.10 (0.01-1.80), 0.120	NE
CC genotype, n (%)	13 (100.0%)	86 (72.3%)	10.46 (0.60-180.9), 0.107	NE
T allele, n (%)	0/26 (-)	35/238 (14.7%)	0.11 (0.01-1.82), 0.122	—
C allele, n (%)	26/26 (100.0%)	203/238 (85.3%)	9.25 (0.55-155.2), 0.122	—
Present smoker, n (%)	0 (-)	13 (10.9%)	0.29 (0.02-5.20), 0.402	NE
CC + present smoker, n (%)	0 (-)	8 (6.7%)	0.49 (0.03-8.90), 0.627	NE
Superficial spreading, n (%)	8 (61.5%)	59 (49.6%)	1.63 (0.50-5.26), 0.416	-
Nodular, n (%)	2 (15.4%)	45 (37.8%)	0.30 (0.06-1.41), 0.127	-
Acral lentiginous, n (%)	0 (-)	5 (4.2%)	0.77 (0.04-14.72), 0.863	-
Lentigo maligna, n (%)	1 (7.7%)	1 (0.8%)	9.83 (0.58-167.4), 0.114	-
Spitzoide, n (%)	0 (-)	5 (4.2%)	0.77 (0.04-14.72), 0.863	-
Nevoid malignant, n (%)	1 (7.7%)	1 (0.8%)	9.83 (0.58-167.4), 0.114	-
Breslow thickness, mm, mean±SD	2.5±2.1	2.0±1.8	-, 0.398	-
Breslow thickness 1.01-2.00 mm, n (%)	2 (15.4%)	36 (30.3%)	0.42 (0.09-1.99), 0.274	-
Breslow thickness 2.01-4.00 mm, n (%)	3 (23.1%)	27 (22.7%)	1.02 (0.26-3.98), 0.975	-
Breslow thickness ⩾4.01 mm, n (%)	3 (23.1%)	15 (12.6%)	2.08 (0.51-8.43), 0.305	-
Mitosis ⩾1 per mm^2, n (%)	7 (53.8%)	78 (65.5%)	0.61 (0.19-1.94), 0.406	-
Regression, n (%)	1 (7.7%)	18 (15.1%)	0.47 (0.06-3.82), 0.478	-
Brisk positive TILs[a], n (%)	4 (30.8%)	35 (29.4%)	1.07 (0.31-3.69), 0.919	-
Microsatellitosis, n (%)	1 (7.7%)	4 (3.4%)	2.40 (0.25-23.20), 0.451	-

Categorical variables were evaluated using ORs and CIs; continuous variables were compared using Student's t test for independent samples. Genotype aORs were not estimable (NE) because all head/neck cases had the CC genotype; adjusted ORs for present smoking and CC plus present smoking were not estimable because no head/neck case was a present smoker. Allele-level models were not fitted. [a]TILs, tumor-infiltrating lymphocytes. Statistically significant estimates are shown in bold; trends are denoted by ^.
